# Amplified Arctic iceberg traffic reshapes benthic biodiversity

**DOI:** 10.1038/s41586-026-10630-4

**Published:** 2026-06-10

**Authors:** Thomas Krumpen, Kirstin S. Meyer-Kaiser, Claudia Wekerle, Lars Ackermann, Deonie Castle, Melanie Bergmann, Mario Hoppmann, Shfaqat A. Khan, Autun Purser, Holger Schmithüsen

**Affiliations:** 1 Alfred Wegener Institute, Helmholtz Centre for Polar and Marine Research, Bremerhaven, Germany; 2 Woods Hole Oceanographic Institution, Woods Hole, MA USA; 3 School of Science, Department of STEM, University of Waikato, Hamilton, New Zealand; 4 DTU Space, Technical University of Denmark, Kongens Lyngby, Denmark

**Keywords:** Cryospheric science, Climate-change impacts, Biodiversity, Climate-change ecology, Ecology

## Abstract

The Arctic is undergoing rapid warming, resulting in retreating sea ice and glaciers^1^, yet how cryospheric changes propagate into the deep ocean remains poorly understood^2^. Here we identify a climate-driven mechanism linking accelerating glacier disintegration to an increase in deep-sea hard-bottom habitats far beyond calving fronts. Seafloor observations in Fram Strait show a localized increase in the density and patchiness of dropstones delivered by debris-laden icebergs. At the same time, four decades of shipboard records show that the occurrence of icebergs increased abruptly in the early 2000s. Backtracking links these icebergs to the main outlet glaciers in northeast Greenland and the Russian High Arctic. In northeast Greenland, the timing of glacier destabilization coincides with this rise, whereas sparse satellite coverage in the Russian sector limits temporal attribution despite indications of enhanced glacier activity. A model sensitivity study shows that, apart from intensified calving, a more dynamic sea ice cover enhances downstream transport of glacial ice. Along these pathways, increased iceberg activity could reshape deep-sea habitats through enhanced melt and associated lithogenic input, and elevate navigational hazards as maritime traffic expands in the Arctic. Although modest compared with the iceberg discharges of Pleistocene Heinrich events, this mechanism provides a modern analogue of long-range cryospheric influence on the seafloor in a warming climate.

## Abstract (web summary)

Accelerated Arctic glacier disintegration and a more dynamic sea ice cover are increasing iceberg-delivered dropstones in the deep ocean, reshaping seafloor habitats and extending cryospheric impacts far beyond glaciers.

## Main

The deep seafloor of the Arctic Ocean remains one of Earth’s least-charted biomes, its physical and biological dynamics concealed beneath a canopy of pack ice. Recent studies, nevertheless, demonstrate that anthropogenic warming and intensified human activity are already imprinting measurable signals at abyssal depths^2,3^. The retreat of sea ice^1,4^ promotes more frequent and extensive ice-edge and under-ice algal blooms^5^, strengthening the biological carbon pump. Diatom-rich aggregates subsequently descend to the seabed, enhancing benthic respiration and stimulating bioturbation^6,7^. As sea ice diminishes, increased human accessibility facilitates industrial exploration, tourism and fishing^8^, with bottom trawling, in particular, inflicting long-lived mechanical disturbance on deep-sea habitats^9^, while mobilizing large pools of sedimentary carbon^10^. Moreover, anthropogenic activities have led to a rise in plastic pollution on the deep Arctic seafloor^11^.

**Fig. 1:**
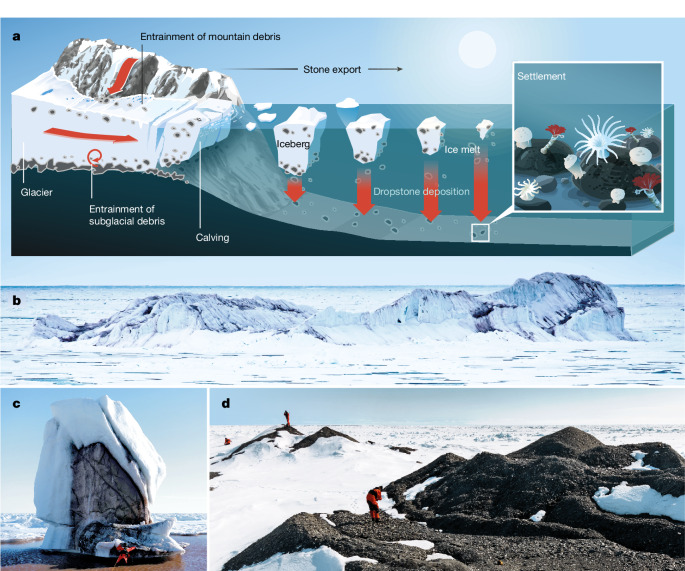
Icebergs transport glacial debris to the deep seafloor. **a**, Debris transported by calved icebergs is released during melt and settles to the seafloor, where the coarser fraction (dropstones) provides a hard substrate for colonizing sessile benthic fauna. **b**,**c**, Iceberg (height 18 m; **b**) and growler with stones and sediments observed in the central Arctic (**c**). **d**, Iceberg embedded in sea ice with surface stones, potentially reflecting supraglacial debris or exposure of debris-rich basal ice, near HAUSGARTEN. The plan area of the exposed stones was about 1,200 m^2^. Credit: **b**,**d**, photographs by M.H.; **c**, photograph by J. Harding, NHK.

By contrast, a slower-acting natural driver of seafloor change is the delivery of glacial ice-rafted debris, particularly its coarse fraction (dropstones), transported by calved icebergs. Once deposited, these erratics become colonization hotspots for hard-bottom fauna^12,13^. Figure 1 provides a conceptual overview of this glacier-to-seafloor pathway. In Fram Strait, the prime gateway between Greenland and Svalbard, a long-term deep-sea monitoring site offers a rare window into these changing abyssal environments. Operated by the Alfred Wegener Institute since 1999, the LTER (Long-Term Ecological Research) observatory HAUSGARTEN^14^ combines autonomous platforms with annual RV *Polarstern* expeditions^15^ to monitor oceanographic, biogeochemical and ecological parameters from the surface to depths of up to 5,500 m.

**Extended Data Fig. 1:**
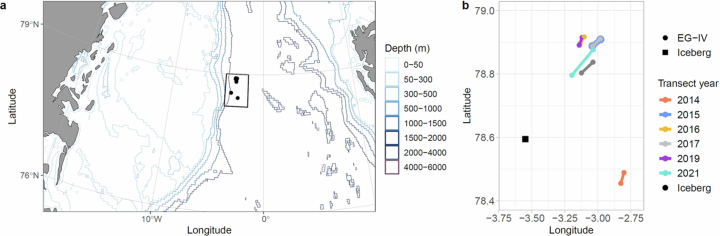
Camera transects at HAUSGARTEN. **a**, location of camera transects at HAUSGARTEN station EG-IV and the iceberg visited in 2021. **b**, Closer view of the area enclosed by the square in panel (**a**), showing the locations of camera transects and the visited iceberg. The 2017 transect aligns with that of 2015.

Recent seafloor imagery from HAUSGARTEN has shown increasing patchiness and higher local densities of dropstones in parts of the Fram Strait abyss, far from any calving front (location is indicated in Extended Data Fig. 1). Whether this reflects a brief episodic pulse or a sustained rise in iceberg traffic driven by accelerating glacial melt remains unclear, as smaller icebergs are difficult to detect in satellite observations and leave no long-term record of their abundance.

In this study, we took a tiered approach by (1) documenting a short-term increase in dropstone density and the associated impact on benthic communities at one HAUSGARTEN station; (2) boarding and sampling a drifting iceberg to characterize its lithogenic cargo; and (3) mining 40 years of RV *Polarstern* visual logs to test for changes in glacial ice frequency across Fram Strait. Finally, for sightings embedded in compact pack ice, we reconstructed trajectories using satellite-derived sea ice-motion data to pinpoint likely source glaciers and assess whether their calving fluxes have accelerated. We then ran high-resolution drift simulations that delineate changing pathways and dispersal corridors for debris-laden glacial ice, demonstrating a lithogenic imprint on deep-sea ecosystems hundreds of kilometres downstream.

## Dropstones shape benthic biodiversity

The presence and distribution of glacially delivered dropstones at HAUSGARTEN station EG-IV on the East Greenland continental margin (about 2,500 m water depth) were documented using a towed deep-sea camera system deployed from RV *Polarstern*. Repeated camera transects were conducted during expeditions in 2015 and 2017 along a 2.6 km track, allowing temporal changes within a defined segment of the seafloor to be assessed over a short timescale (Methods and Extended Data Fig. 1).

**Fig. 2:**
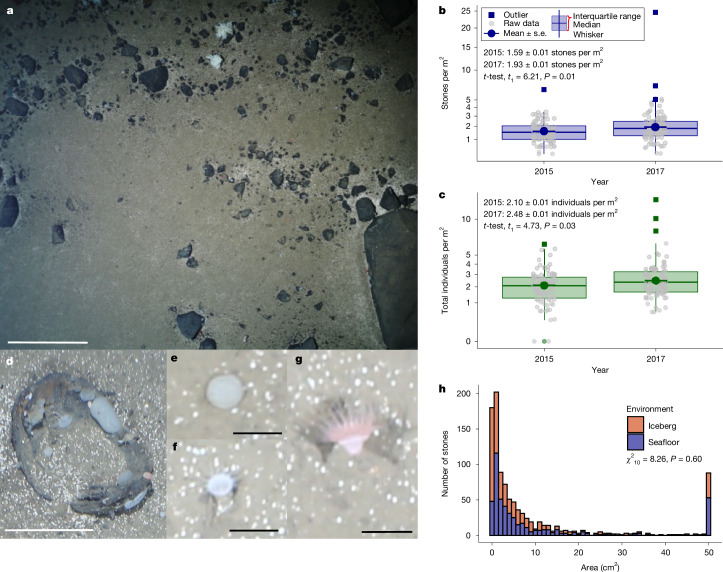
Dropstones enhance seafloor habitat heterogeneity and biodiversity. **a**, Photograph of a large dropstone patch at HAUSGARTEN station EG-IV (2,500 m depth, recorded in 2021), with stones 1.2–325 cm^2^. **b**, Boxplot of stone densities in the repeated transect in 2015 and 2017. Shown are raw data and outliers (see legend), together with the mean, median, interquartile range and whiskers with length 1.5× the interquartile range. The indicated values represent mean ± s.e. **c**, Boxplot of the density of dropstone fauna in the repeated transect in 2015 and 2017. **d**–**g**, Common dropstone taxa: unidentified sponges (**d**); *Tentorium semisuberites* (**e**); *Bathyphellia margaritacea* (**f**); *Amphianthus* sp (**g**). **h**, Size distributions of stones on the iceberg sampled in 2021 and on the seafloor nearby (station EG-IV, 2,500 m depth). Scale bars, 50 cm (**a**); 10 cm (**d**); 2 cm (**e**–**g**).

Image analysis revealed clustered deposition of newly delivered, predominantly small dropstones consistent with melt-out from passing icebergs. Dropstone density in 2017 (1.93 ± 0.01 stones per m^2^, mean ± s.e.) was significantly higher than in 2015 (1.59 ± 0.01) (*t*_1_ = 6.21, *P* = 0.01) (Fig. 2b), whereas the size distribution of stones differed, and mean stone area decreased from 7.92 ± 1.70 cm^2^ in 2015 to 4.25 ± 1.96 cm^2^ in 2017 (*χ*^2^ test, *P* = 0.003). The distribution of dropstones on the seafloor did not deviate from randomness in 2015 (*χ*^2^ test, *P* = 0.99), but in 2017, stones were significantly clumped (Morisita index = 1.13, *χ*^2^ test, *P* < 0.001). To avoid bias from a small number of localized stone clusters, images with exceptionally high stone densities were excluded from these analyses. A similar increase in small dropstones (<6.4 cm^2^) was reported in the eastern Fram Strait between 2011 and 2015 (ref. ^11^), suggesting that the observed pattern may extend beyond the single station surveyed here.

Dropstones function as hard-bottom habitats and offer settlement substrata for fauna dependent on solid surfaces^13,16^. A higher density of dropstones on the seafloor promotes biodiversity by increasing the number and spatial variability of attachment sites available to sessile organisms^12,17^. The most common taxa inhabiting dropstones on the East Greenland margin across all transects were sponges and cnidarians, including *Tentorium semisuberites*, *Bathyphellia margaritacea* and *Amphianthus* sp. (Fig. 2d–g). Consequently, the increase in stone densities was reflected in significantly higher densities and richness of dropstone-associated fauna in 2017 compared with 2015 (Fig. 2c, density: *t*_1_ = 4.73, *P* = 0.03; richness: *t*_1_ = 5.94, *P* = 0.01, images with high stone densities were excluded). Although newly deposited stones were predominantly smaller, previous work in Fram Strait reported no differences in community composition among dropstones of varying sizes^12^, suggesting that the observed size differences are unlikely to influence benthic community composition. Importantly, we observed significant increases in the densities of two encrusting sponge morphotypes between 2015 and 2017 (*t*-test, blue sponge, *t*_1_ = 7.6, *P* = 0.006; bubble sponge, *t*_1_ = 9.7, *P* = 0.002). In 2015, all dropstone-associated taxa were randomly distributed (*χ*² test, *P* > 0.05), whereas in 2017, two encrusting morphotypes (likely sponges or bryozoans) and a serpulid polychaete species showed significantly clumped distributions that mirrored the dropstone pattern (*χ*² test, *P* < 0.05). Serpulid polychaetes are among the first metazoans to settle on hard-bottom habitats in Fram Strait^18^. Other early colonists of Arctic hard-bottom habitats include hydrozoans and foraminiferans^19,20^, although these taxa were too small to be detected by the Ocean Floor Observation System (OFOS).

The availability of hard-bottom dropstone habitats is one of the primary drivers of community composition in the deep Arctic Ocean, and its influence extends far beyond the stones themselves^16^. Hard-bottom taxa such as sponges, anemones and soft corals, in turn, serve as basibionts for epibiotic shrimps, crinoids and amphipods^12,13^. Dropstones interrupt laminar flow across the sediment, and the turbulent eddies that result provide heterogeneous habitats that increase meiofaunal diversity^21^. Although empty niche space on newly deposited dropstones is rapidly exploited by early-successional opportunistic species, the assembly of mature communities in polar environments can take decades^18,22^.

## Rising iceberg frequencies in Fram Strait

**Extended Data Fig. 2:**
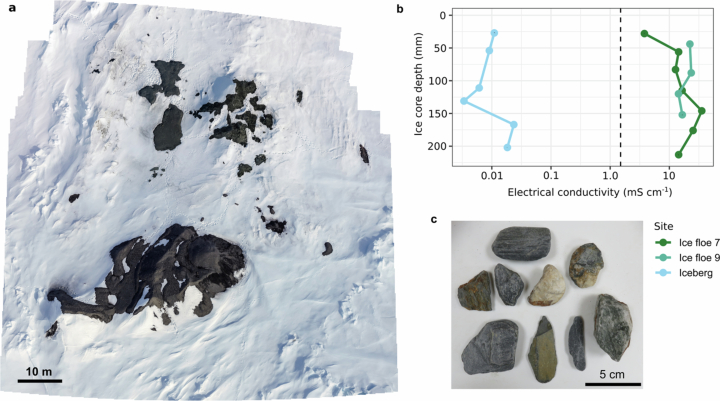
Evidence for lithogenic material transport by icebergs. **a**, Nadir-looking aerial photomosaic of a small iceberg accessed by helicopter from RV *Polarstern* in 2021 (see also Fig. 1d), showing dark mounds of shale and quartz stones; **b**, electrical conductivity measurements made from ice cores collected at the iceberg and two nearby sea ice floes; dotted line divides freshwater and saltwater origin values; **c**, Photo of stones collected from the visited iceberg in 2021.

In 2021, we had the rare opportunity to visit a small iceberg drifting in the vicinity of the HAUSGARTEN observatory and collect and analyse stones exposed at its surface (Fig. 1d and Extended Data Fig. 2a). These stones formed distinct mounds protruding 1–5 m above the snow, and may either represent supraglacial debris from the parent glacier or debris-rich basal ice exposed after the iceberg overturned. This vertical structuring of lithic material within icebergs plays an important role in controlling where and when it is released during drift (for example, refs. ^23–25^).

Similarities between stones on the iceberg and the seafloor in 2021 indicate a direct link between ice-rafted material and seafloor dropstones. Stones in both environments were identified as shale and quartz and exhibited right-skewed size distributions that did not differ significantly (*χ*²_10_ = 8.26, *P* = 0.60) (Fig. 2h). The smallest size category (<1 cm^2^) was underrepresented in seafloor images, likely as a consequence of the towed camera resolution or burial by sediments. The glacial origin of the iceberg was confirmed by its very low salinity: the electrical conductivity of an ice core was just 0.003–0.023 mS cm^−1^, about three orders of magnitude lower than that of the nearby sea ice floes (Extended Data Fig. 2b).

To examine whether the locally observed increase in dropstone density and associated biodiversity represents random variation or follows a systematic trend, we assessed changes in iceberg frequency within the Fram Strait region over time. However, detecting icebergs enclosed in dense pack ice is inherently difficult, as satellite sensors can resolve only very large icebergs and often fail to distinguish smaller fragments from surrounding sea ice^26,27^. Consequently, long-term records of iceberg frequency, variability or drift pathways in the Arctic are virtually absent.

**Fig. 3:**
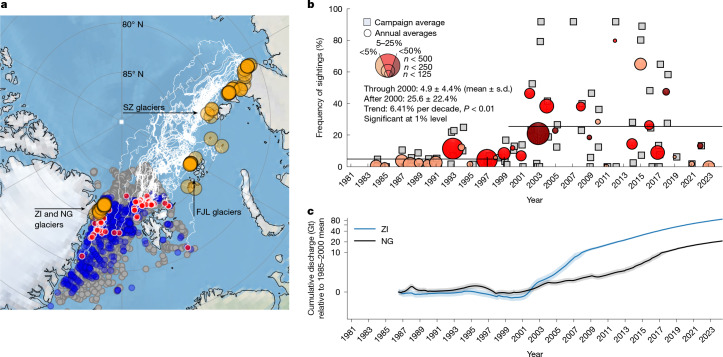
Rising iceberg occurrence links to Arctic glacier discharge. **a**, Locations of visual synoptic observations conducted aboard RV *Polarstern* between 1981 and 2024. The grey dots show sites without iceberg sightings, whereas the blue dots indicate locations where icebergs, growlers or bergy bits were observed. A subset of sighted icebergs (red dots) could be backtracked using a Lagrangian drift model (white trajectories) to their potential source regions (orange circles): the NG and ZI glacier, and glaciers located on SZ and FJL. **b**, Frequency of iceberg sightings during individual *Polarstern* expeditions (squares, campaign averages; circles, annual means). The circle size denotes the number of synoptic observations contributing to the campaign mean. The circle colour represents the proportion of sightings containing more than five individual icebergs. The black lines indicate mean sighting frequencies before 2000 and thereafter. **c**, Cumulative glacier discharge (relative to 1985–2000 mean, in gigatonnes (Gt)) of the ZI (blue) and NG (black), derived from satellite and airborne altimetry data (source, ref. ^48^).

**Extended Data Fig. 3:**
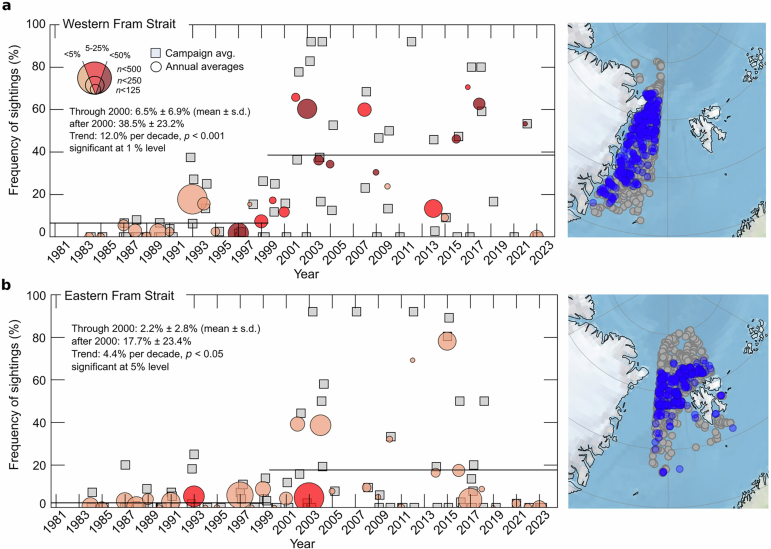
Rising iceberg occurrence in western and eastern Fram Strait. **a**,**b**, Frequency of iceberg sightings during individual RV *Polarstern* expeditions (squares: campaign averages; circles: annual means) for western (**a**) and eastern (**b**) Fram Strait. Circle size denotes the number of synoptic observations contributing to the campaign mean. Circle color represents the proportion of sightings containing more than five individual icebergs. Black lines indicate mean sighting frequencies prior to 2000 and thereafter.

To quantify glacial ice frequencies within Fram Strait, we therefore relied on direct visual sightings rather than satellite observations. These sightings were recorded every 3 h aboard the RV *Polarstern* during approximately 99 expeditions conducted over the past 40 years (Methods). Figure 3a shows the spatial distribution of these visual observations, distinguishing between transects with and without glacial ice encounters. Figure 3b shows iceberg frequencies for individual expeditions and annual averages within the region bounded by 70°–85° N, 30° W–30° E. Our analysis showed a strong increase in iceberg frequency of approximately 6.4% per decade (*t*-test, *P* = 0.01). This trend shows an almost stepwise pattern, with low frequencies (4.9 ± 4.4%) observed before 2000 and higher frequencies (25.6 ± 22.4%) thereafter. Moreover, the proportion of iceberg groups containing more than five individual icebergs increased by 4.5% per decade (*t*-test, *P* = 0.017), both trends being statistically significant. Importantly, these positive trends persist even when the region is subdivided and the western and eastern Fram Strait are considered separately (for example, using a regional split at 5° W; Extended Data Fig. 3). Uncertainties associated with the visual observation method, such as variations in visibility and the potential underrepresentation of smaller growlers and bergy bits, are discussed in detail in the Methods. Despite these limitations, the density and temporal continuity of the observations provide a robust basis for detecting relative changes in glacial ice occurrence.

The increase in iceberg sightings in Fram Strait implies a concurrent intensification of calving activity at upstream source glaciers. To identify iceberg origins, we applied a satellite-based Lagrangian backtracking approach. Trajectories were reconstructed only for sightings embedded in compact pack ice, requiring sea ice concentration along the back-trajectory to remain ≥80%, with no more than 30 cumulative days below this threshold (Methods). In total, 106 of 1,362 iceberg sightings met these criteria for trajectory reconstruction (Fig. 3a, red circles). The resulting trajectories (white lines) indicate that icebergs observed in Fram Strait predominantly originate from two distinct regions. Sightings west of 5° W trace back to northeast Greenland, where the marine-terminating Nioghalvfjerdsfjorden glacier (NG) and Zachariæ Isstrøm (ZI) represent the most probable sources. By contrast, iceberg sightings east of 5° W (including HAUSGARTEN) originate primarily from marine-terminating glaciers in the Russian Arctic, particularly those located on Severnaya Zemlya (SZ) and Franz Josef Land (FJL). However, sightings in the eastern Fram Strait were less frequently traceable owing to the more dynamic and fragmented sea ice conditions in this region^28^. A few reconstructed trajectories also point towards the mainland south of SZ, where no marine-terminating glaciers exist. These cases most likely reflect uncertainties in the backtracking, particularly for longer travel times and near the ice-margin regions^29^.

## Iceberg rise tracks glacier change

**Extended Data Fig. 4:**
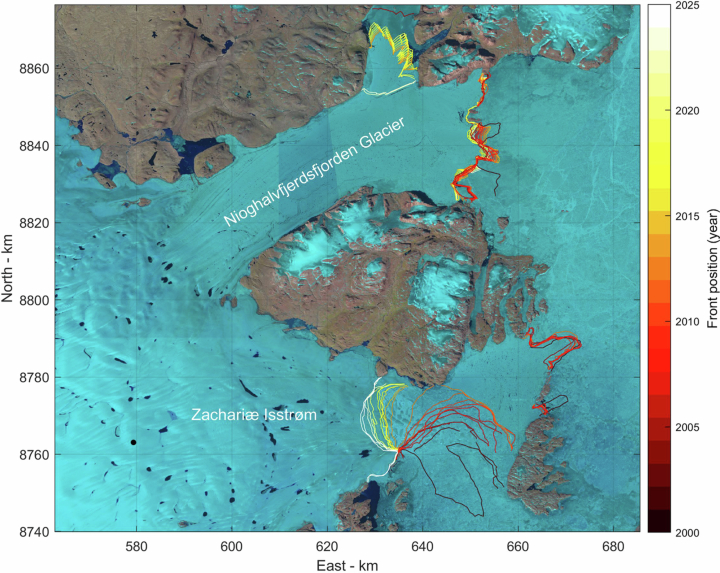
Glacier frontal positions mapped from 2000 to 2025. Frontal positions from 2000 to 2025 based on Landsat 5–8 optical satellite imagery^32^. The Landsat 8 satellite image (surface reflectance) shown in the background was captured on August 29, 2025, and obtained from the website https://glovis.usgs.gov/app (United States Geological Survey).

Back-trajectories identify the Northeast Greenland Ice Stream (NEGIS), with its main outlets NG and ZI (Extended Data Fig. 4), as the principal source region for icebergs entering the western Fram Strait. An analysis of NEGIS discharge rates based on satellite and altimetry data shows a sustained stepwise increase beginning around 2003 (Fig. 3c). The timing of this shift aligns closely with the observed increase in iceberg frequency in the Fram Strait. The shift has been interpreted as marking the end of a decades-long quasi-stable regime and the onset of increased dynamic discharge from the NEGIS outlets^30^.

Within the NEGIS sector, ZI provides the clearest and best-resolved link to the iceberg frequencies in Fram Strait. Many studies document the loss of the former stability of ZI following years of progressive ice-shelf thinning, with rapid acceleration and retreat after the year 2000 (refs. ^31,32^) that led to massive ice loss at the calving front (compare Fig. 3c and Extended Data Fig. 4). This rapid collapse was triggered by regional atmospheric and oceanic warming, which raised fjord-water and surface air temperatures, reduced mélange and sea ice buttressing, and thereby increased the susceptibility of the glacier to calving^33^. The neighbouring glacier, NG, which has a broad, buttressed floating tongue approximately 80 km long, also releases large icebergs downstream. However, its increase in ice discharge rate after 2000 was less pronounced, most likely because the tongue was mechanically stabilized by persistent sea ice and mélange cover during this period^32–34^.

Although back-trajectories indicate that NEGIS outlets primarily drive the increase in iceberg sightings in the western Fram Strait, the eastern Fram Strait signal is most likely linked to marine-terminating glaciers on SZ and FJL. Since the early 2000s, glaciers and ice caps across these archipelagos have also undergone sustained thinning and frontal retreat, accompanied by a pronounced increase in mass loss that approximately doubled relative to pre-2010–2011 conditions^35–39^.

**Fig. 4:**
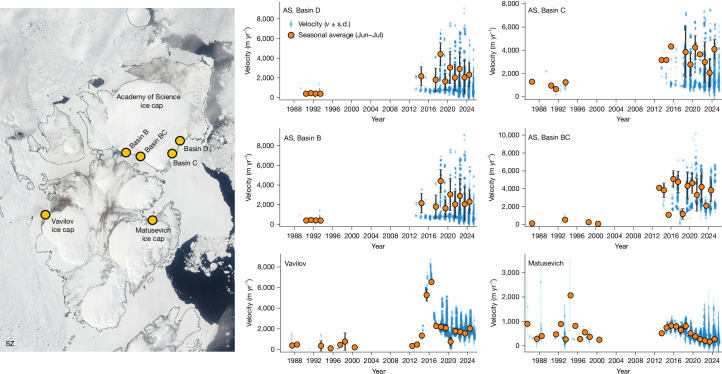
Surface velocities indicate dynamic change on SZ. Surface velocity time series for selected marine-terminating glaciers and ice-cap outlets on SZ. Individual ITS_LIVE velocity^48^ (*v*) observations derived from Landsat image pairs are shown with associated measurement uncertainties (blue symbols with vertical bars, m yr^−1^ ± s.d.). Seasonal mean velocities (orange symbols) represent time-weighted averages of observations acquired within ±30 days around 1 July of each year. The satellite image shown on the left was captured by Terra/MODIS on 18 June 2025 and made available through the NASA Worldview application (https://worldview.earthdata.nasa.gov).

Compared with northeast Greenoubled relative to pre-2010–2011 conditions, surface velocity and mass change on SZ and FJL, particularly before 2010, are sparse and fragmented. This limits our ability to directly link the early-2000s stepwise increase in iceberg frequency to the destabilization of specific marine-terminating glaciers in the Russian High Arctic. An analysis of satellite-derived surface velocities from the ITS_LIVE archive for representative marine-terminating outlets on SZ and FJL underscores this limitation (Fig. 4 and Extended Data Fig. 8). The sparse and irregular temporal coverage of the satellite data, especially during the early-2000s transition period, precludes robust trend analysis and prevents reliable detection of short-lived extreme events or regime shifts. Nevertheless, several outlets on SZ, particularly on the Academy of Sciences Ice Cap (AS), exhibit higher flow speeds after around 2012 compared with the pre-2000 period, consistent with enhanced dynamic discharge and calving. Another striking example of dynamic change on SZ is the Vavilov Ice Cap, which experienced a well-documented episode of rapid reorganization and strong mass loss after 2013 (Fig. 4; refs. ^37,40^). By contrast, the available ITS_LIVE records for FJL (Extended Data Fig. 8) do not show a comparable acceleration signal.

Importantly, calving from SZ or FJL does not translate directly into immediate export towards Fram Strait. Much of the glacial ice released into the sea can remain mechanically locked within the landfast-ice belt for months or years, with release occurring episodically during fast-ice break-up and periods of enhanced pack mobility^41^. Such storage and episodic releases are expected to temporally smear any source-side acceleration signal, thereby complicating the link between glacier change and the stepwise increase in Fram Strait iceberg sightings. In this context, the available velocity record suggests that SZ is the more likely of the two Russian sectors to have contributed to the enhanced eastern Fram Strait iceberg occurrence, whereas a distinct dynamical signal from FJL is not evident.

Beyond these primary source regions, icebergs from the Canadian High Arctic could, in principle, reach Fram Strait through the Beaufort Gyre and subsequent entrainment into the Transpolar Drift, although such long-range drift towards the Eurasian Arctic appears to be rare^42,43^. To assess whether this contribution has changed over time, we backtracked sea ice exiting the Arctic through a virtual gate at 83° N (25° W–25° E) over three decades. This analysis, however, shows no post-2005 rise in the fraction of Beaufort-origin ice reaching Fram Strait (Methods and Extended Data Fig. 9). Accordingly, an enhanced contribution of Canadian icebergs is unlikely to explain the observed stepwise increase.

## Faster drift under reduced sea ice

Although the timing of the iceberg increase in the western Fram Strait aligns with changing discharge from the NEGIS outlets, the rise in iceberg occurrence in the eastern Fram Strait is not readily attributable to individual glaciers and instead seems to reflect a mixed signal, to which changes in iceberg drift dynamics may also have contributed, albeit with a smaller magnitude.

To examine the dynamic component, we performed a sensitivity experiment with the Finite-VolumE Sea Ice-Ocean Model (FESOM2) coupled to an interactive iceberg module (Methods). Over a 38-year simulation period, icebergs at a constant calving rate were released throughout the year from glaciers in the identified source regions. This configuration is not intended to reproduce the absolute number of icebergs in the Arctic, but rather to isolate the dynamics of drifting icebergs and the processes acting along their trajectories across different phases of the simulation.

**Fig. 5:**
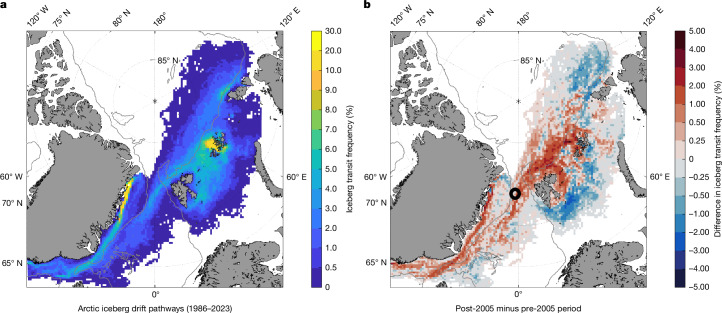
Iceberg transport intensified along major drift corridors. **a**, Simulated iceberg transit frequency (relative frequency of iceberg passages through each grid cell) for icebergs released from NG, ZI, SZ and FJL between 1986 and 2023. Two dominant export corridors emerge: a western pathway originating from the NG and ZI glacier and flowing southwards along the East Greenland coast, and an eastern pathway connecting SZ and FJL with the central and eastern Fram Strait. **b**, Difference in transit frequency between the later (post-2005) and earlier (pre-2005) periods. Positive values (red) along both corridors indicate more efficient export towards the Fram Strait, whereas negative values (blue) in the Barents and Laptev seas reflect earlier melt under reduced sea ice cover. The black circle shows HAUSGARTEN station EG-IV.

Model results show two persistent drift corridors linking the upstream glacier sectors to Fram Strait (Fig. 5a). Icebergs released from NG and ZI are transported primarily along the East Greenland margin and enter the western Fram Strait, whereas icebergs originating from SZ and FJL are advected across the central Arctic and exit through the central and eastern Fram Strait. This spatial structure closely matches the sector-scale source attribution obtained from the satellite-based backtracking analysis and therefore provides independent support for the two dominant export pathways.

**Fig. 6:**
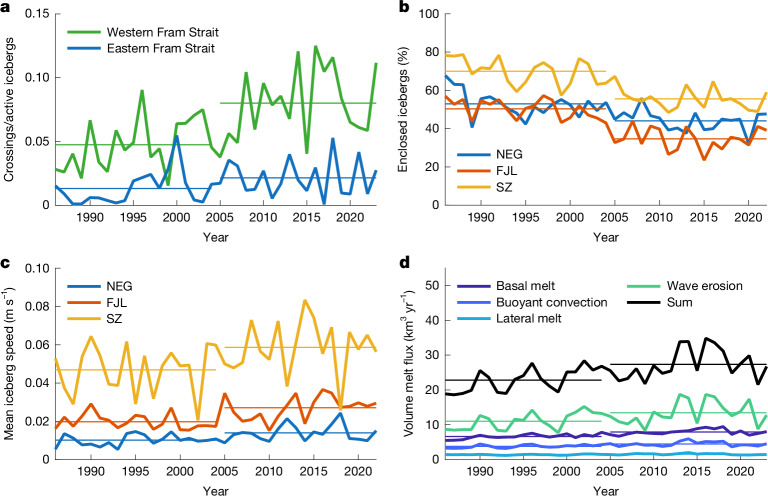
Faster drift of less constrained icebergs promotes enhanced melt. **a**, Annual fraction of simulated icebergs crossing virtual gates in the western (78° N,25° W–5° W) and eastern (80° N, 5° W–20° E) Fram Strait (number of crossings relative to the number of active icebergs). **b**, Mean fraction of simulated icebergs enclosed within dense pack ice during the first year after calving, shown for trajectories originating from NEG, FJL and SZ. **c**, Mean iceberg drift speed during the first year after calving for trajectories originating from NEG, FJL and SZ. **d**, Annual iceberg volume melt flux integrated over all active icebergs in the simulation. The colours show individual melt components (basal melt, buoyant convection, lateral melt and wave erosion), and the black line indicates the total melt flux. The horizontal lines indicate the mean for the periods 1986–2004 and 2005–2023. The mean and s.d. values are provided in Extended Data Table 1.

Comparing simulated iceberg transit frequencies before and after the stepwise increase around 2005 indicates that both principal pathways experienced a modest but spatially coherent rise in the later period. Although small in absolute terms (a few percentage points), the increase corresponds to a relative gain of about 56% in the eastern and about 69% in the western Fram Strait compared with baseline frequencies during 1986–2004 (Fig. 6a). This suggests that higher Fram Strait iceberg frequencies reflect not only increased upstream calving but also more efficient downstream transport.

The increase in the number of icebergs crossing the western and eastern Fram Strait, together with rising mean drift speeds for icebergs originating from NG, ZI, SZ and FJL, is shown in Fig. 6a,c. The reduced residence time of icebergs reflects the increasing mobility of the surrounding Arctic pack-ice cover, a trend documented in satellite observations^28,44,45^ and reproduced by the model. Concurrently, the retreating and more dynamic sea ice exposes a larger fraction of icebergs to open-water conditions and winds, reducing the frequency with which they remain embedded in dense pack ice (Fig. 6b). As a consequence, the simulations show increasing iceberg melt rates, as oceanic warming and wave-induced melt processes become more effective (Fig. 6d). Together with the pronounced summer sea ice retreat in the Barents and Laptev seas, this leads to a declining iceberg occurrence in these regions (Fig. 5b, blue shading).

## Dispersal pathways of iceberg debris

We expect that the dispersal of lithogenic material transported by icebergs follows the main drift corridors shown in Fig. 5. The HAUSGARTEN station EG-IV lies directly within the eastern pathway, suggesting that Russian glaciers are a plausible source of the observed dropstone clustering on the seafloor. However, a more precise quantification of dropstone deposition remains challenging because our model does not explicitly simulate lithogenic cargo and its release. Dedicated cargo-enabled iceberg models can address this gap, yet their predictive skill still depends on poorly constrained properties of iceberg cargo, including the total lithogenic load, its vertical distribution within the ice, and how these vary across source regions. Observational constraints from Greenland indicate strong heterogeneity in debris-rich iceberg ice (0.1–45% by mass; median 3.48%; ref. ^25^) and suggest that cargo release is often biased towards early melt-out of basal layers close to the calving front rather than hundreds of kilometres downstream. Comparable constraints for the Russian High Arctic sources are currently lacking.

As glacier mass loss accelerates in Greenland and the Russian High Arctic, the number of icebergs entering the corridors outlined in Fig. 5 is likely to increase. This suggests a sustained and potentially increasing supply of hard-bottom substrate to deep-sea ecosystems, with the discussed long-term implications for habitat structure, colonization and community assembly. The rapidly increasing iceberg traffic also poses a greater navigational risk across Fram Strait, as collision hazards increase along the East Greenland margin and within the Transpolar Drift outflow. These findings underscore the importance of enhanced monitoring and dynamic hazard forecasting for shipping, fishing and offshore operations.

## Conclusion

Together, our results indicate a direct, climate-driven connection between glacier change at the surface, amplified iceberg traffic, and the increased availability of hard-bottom habitats on the deep seafloor. The impact of accelerated calving at marine-terminating glaciers is thus not confined to coastal environments; through predictable and increasingly dynamic drift pathways, it leaves a lasting lithogenic imprint on the deep Arctic habitats far from the calving front. As the cryosphere continues to warm, increasing iceberg discharge and drift velocity intensify this imprint and are expected to further amplify habitat heterogeneity and biodiversity along these major drift pathways. However, the assembly of a climax community on newly deposited dropstones takes decades^18,24^. Although these emerging pathways are far smaller in magnitude than the massive iceberg discharges of Pleistocene Heinrich events^46,47^, they represent a modern, climate-driven analogue in which enhanced calving drives the dispersal of lithogenic material across the Arctic Ocean.

## Methods

### Iceberg imaging and sampling

During expedition PS126 of RV *Polarstern*, the opportunity arose to visit a large iceberg in the vicinity of HAUSGARTEN carrying numerous dark stones. The iceberg was accessed by helicopter from the ship on 14 June 2021 at the coordinates 78° 35.66′ N, 3° 32.92′ W, allowing us to collect samples of the transported stone material.

A series of overlapping, downward-facing images of the iceberg and its stone load were recorded from the helicopter at an altitude of about 100 m using a Canon EOS digital camera with a lens operated at about 50 mm. The images were stitched together using Agisoft Photoscan Pro. Stone piles placed approximately 5 m apart by field personnel served as a size reference for both individual images and the final mosaic (Extended Data Fig. 2a). Additional close-range images of stone clusters were acquired on the iceberg using a Nikon D850 with a 50 mm lens, with a ruler included in each frame for scale. The planar area of 20 randomly selected stones per image (*n* = 500 in total) was measured using ImageJ. Furthermore, stones were sampled to determine their mineralogical composition. Representative specimens spanning the observed morphological range were collected for classification (Extended Data Fig. 2c).

An ice core (9 cm in diameter) was collected from the upper 2 m of the iceberg (after snow removal) using a hand auger with an electric drill (Kovacs Enterprise). The coring location was about 50 m away from the nearest stone pile to avoid stone interference during drilling. Three replicate measurements of electrical conductivity were made from sub-cores 27–57 cm long (*n* = 6) to determine the salinity of the ice. Identical methods were used to assess the salinity of two sea ice floes nearby for reference (78° 54.11′ N, 3° 9.39′ W and 79° 01.44′ N, 5° 42.49′ W).

### Seafloor observations

Images were recorded from the seafloor at HAUSGARTEN station EG-IV (2,500 m depth) using either the OFOS or the Ocean Floor Observation and Bathymetry System between 2015 and 2021 (ref. ^49^). The exact transect location varied by year due to ice conditions (Extended Data Fig. 1b). In 2015 and 2017, the same transect was surveyed, allowing for a comparison between years in an area of seafloor about 5,400 m^2^. OFOS included a downward-facing camera (Canon EOS), strobes that flashed to illuminate each image and downward-facing laser points that served as a size scale for the image and stone plan area. Telemetry data showed distance to the seafloor, and all images were recorded at a target altitude of 1.5 m. OFOS images were recorded automatically every 30 s while the ship transited at 0.5 knots. Images that were too bright, too dark or at an anomalous altitude (that is, outside the range of 1.3– 1.6 m) were considered ineligible for analysis.

We observed glacial dropstones on the seafloor in OFOS images, ranging from 0.6 cm to 73 cm. The vast majority of stones on the deep seafloor north of 45° N are glacial in origin^50^. Near-bottom currents in the bathyal Fram Strait are <10 cm s^−1^, not fast enough to uncover buried stones or bedrock^14,21^.

An exception occurs at a steep reef in the eastern Fram Strait, where stronger near-bottom currents expose bedrock and mobilize locally derived stones^51^. However, this feature lies several hundred kilometres from station EG-IV, where our imagery was collected. The stones observed on both the seafloor and the visited iceberg consist of shale and quartz (Extended Data Fig. 2). Taken together, the geological, bathymetric and oceanographic context indicates that stones observed on the seafloor at station EG-IV represent glacial dropstones.

The densities of stones in 30 randomly selected images from the beginning, middle and end of the transect were calculated each year (total of 90 images per year). Dropstones and dropstone-associated fauna were annotated in each image using the online tool BIIGLE (Bio-Image Indexing and Graphical Labelling Environment; biigle.de; ref. ^52^). OFOS images recorded at 3 m altitude reliably show seafloor features and fauna >1 cm across^49^. Images in this study were recorded at about 1.5 m altitude, allowing for visualization of fauna and features to 0.6 cm across. Fauna visible in our OFOS images included sponges, bryozoans, tunicates, anemones, soft corals, serpulid polychaetes, barnacles, sea stars and crinoids. Some taxa could be identified based on taxonomic voucher samples collected in previous studies throughout Fram Strait^53^. Taxa for which no voucher had been identified were given morphotype descriptors. The densities of stones and of each dropstone-associated species in 2015 and 2017 were calculated by dividing the number in each image by the planar area of that image. Plan areas of 250 stones in randomly selected 2015 and 2017 images (*n* = 250 per year) were measured using the rectangle select tool in BIIGLE. Plan areas of 500 stones from randomly selected 2021 images were measured using the straight line tool for measurement of length and width in ImageJ.

Dropstones are typically deposited on the seafloor in clusters, and slight variations in the swing of the OFOS camera could cause these clusters to appear in one year but not another. To avoid biasing the results by a small number of images containing dense stone clusters, we removed outlier images with anomalously high stone densities (>5 stones per m^2^) before analysis (*n* = 1 image, 2015; *n* = 3 images, 2017). Differences in univariate metrics (that is, stone density, fauna density) were evaluated using parametric analysis of variance (ANOVA) tests or non-parametric Kruskal–Wallis tests, when the assumptions of ANOVA were violated. Chi-square tests were used to test for differences in the size distributions of stones on the iceberg and on the seafloor and between years (2015 and 2017). Statistical analyses were conducted in the R environment using the packages vegan, pairwiseAdonis and ggplot2.

### Ship-based glacial ice observations

Small icebergs, growlers and bergy bits generally evade detection by satellite imagery, necessitating visual observations for monitoring their presence. On board the research vessel *Polarstern*, iceberg observations have been routinely carried out every 3 h by the German Weather Service (DWD). These observations are part of the Surface Synoptic Observations, a globally standardized meteorological data collection program maintained according to the guidelines set by the World Meteorological Organization (WMO). This continuous observational practice has been operational since the commissioning of *Polarstern* in 1984, providing essential information for onboard activities such as navigation, route planning and aircraft operations, while simultaneously contributing to global meteorological networks.

**Extended Data Fig. 5:**
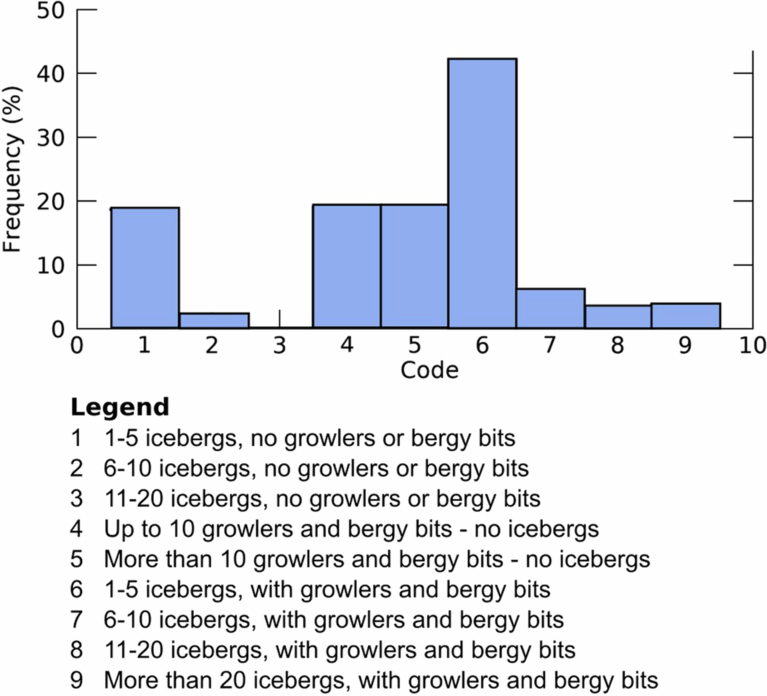
Observed frequencies of different iceberg classes. Shown are the average frequencies (%) at which each glacial ice class (WMO 2019-2025 alphanumeric table 0439/ BUFR table 020035) was recorded across all expeditions over the past 40 years. Definitions of each class are provided in the legend.

Synoptic observations from ships include key atmospheric parameters, current weather conditions and basic oceanic parameters, such as air and water temperature, air pressure, wind speed and direction, humidity, precipitation, cloud cover and cloud types, visibility, wave height and periodicity, all encoded in a globally standardized reporting format ‘FM 13 SHIP’^54^. In ice-covered waters, observations include details on the ice-going ability of the vessel, the type and developmental stage of encountered sea ice, and sightings of glacial ice classified according to WMO^54^ alphanumeric table 0439/BUFR table 020035. For glacial ice, this code specifically describes the type (icebergs, bergy bits and growlers) and quantity of observed glacial ice, reported in increments of five (see legend in Extended Data Fig. 5). The absence of glacial ice and the inability to perform observations due to restricted visibility conditions are also explicitly noted.

The processing and analysis of glacial ice observations were conducted as follows. Initially, all synoptic records collected between 1981 and 2024 were compiled separately by campaign (data for individual campaigns are accessible through PANGAEA^55^). Subsequently, all valid glacial ice observations within the Fram Strait region (70°–85° N, 30° W–30° E) were filtered. Observations were defined as valid if visibility exceeded 1 km. This resulted in approximately 8,200 valid observations, with glacial ice reported around the vessel in about 32% of these cases. The spatial distribution of observations is shown in Fig. 3a. Note that repeated sightings at the same position or within a radius smaller than the visibility at the time of observation were excluded to avoid double-counting.

Following this filtering, campaign-specific and annual mean frequencies of iceberg sightings were calculated (Fig. 3b), as well as frequency distributions of individual glacial ice types. Extended Data Fig. 5 shows the frequencies of glacial ice observations, along with the corresponding classification codes. Most frequently observed were 1–5 icebergs accompanied by growlers and bergy bits (class 6), followed by occurrences of only icebergs (1–5, class 1) or only growlers and bergy bits (class 4).

**Extended Data Fig. 6:**
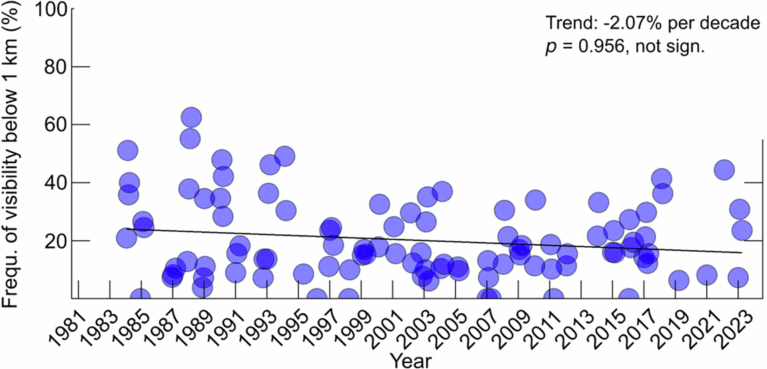
Frequency of low-visibility conditions during individual expeditions. Shown is the proportion of synoptic observations aboard RV *Polarstern* that could not be conducted due to limited visibility (below 1 km) during individual expeditions (blue circles). Visibility may be restricted by snow, rain, fog, or similar factors. A trendline is plotted on top.

Visual glacial ice observations inherently involve uncertainties affecting their representativeness. First, sightings may vary among individual observers, particularly under challenging visibility conditions or low sun angles. Moreover, observer performance might evolve over time, for example, as observers gain experience. Although this subjective error is difficult to quantify precisely, it is considered small because of the consistent use of trained meteorological experts. Second, weather conditions such as precipitation or fog can limit the number of visual observations carried out during expeditions. On average, about 20.4% of planned observations are hindered by visibility conditions below the 1 km threshold, although this percentage can be considerably higher for individual expeditions. However, analysis of the 40-year observational record shows no significant trend in visibility conditions, indicating that no systematic long-term biases are present in the observational time series (Extended Data Fig. 6). Third, larger and more prominent icebergs are proportionally detected more frequently than smaller growlers and bergy bits. This uncertainty also remains difficult to precisely quantify.

Given these limitations, definitive quantification of absolute iceberg frequencies, interannual variability or spatial distribution patterns remains uncertain and should be interpreted cautiously. Nevertheless, Fram Strait, and specifically the HAUSGARTEN region, benefits from frequent observation efforts, on average traversed by the AWI 2.5 times per year, yielding a continuous and extensive dataset. The density and duration of this observational time series enable robust detection of relative changes in glacial ice occurrence despite the discussed observational constraints.

### Backtracking of glacial ice

To identify the source regions (calving sites) of glacial ice and, consequently, the origin of stones deposited on the deep seafloor, we backtracked ship-based glacial ice observations using satellite-derived ice drift data. For this purpose, we used IceTrack^56^, a tool originally developed for backtracking sea ice to the sites where it was formed.

For the backtracking approach, ice is traced backwards on a daily basis from observed locations using three independent low-resolution sea ice drift products: (1) OSI-405-c ice-motion data from the Ocean and Sea Ice (OSI) Satellite Application Facility^57^ (SAF); (2) MERGED ice-motion vectors provided by the Center for Satellite Exploitation and Research^58^ (CERSAT); and (3) Polar Pathfinder daily motion vectors (v.4.1) available from the National Snow and Ice Data Center^59^ (NSIDC). Backtracking is terminated whenever trajectories intersect coastlines, encounter fast-ice edges, or when the satellite-derived ice concentration (from OSI SAF products OSI-430-a and OSI-450-a) falls below 50%.

This Lagrangian tracking method is applicable to glacial ice as long as icebergs, growlers and bergy bits are firmly embedded within the pack ice and carried along by it. Under looser ice-pack conditions or for deep-drafted icebergs, the validity of this assumption diminishes. Here, ocean currents begin to strongly influence the drift, causing icebergs to diverge from the general motion of the surrounding, mainly wind-driven, sea ice^60^. To account for this limitation, we restricted backtracking to sightings of glacial ice firmly entrained within the pack ice at the time of observation and throughout their drift. Specifically, we imposed a criterion that satellite-derived ice concentration along each trajectory should not fall below 80% for more than 30 cumulative days. A total of 106 out of the 1,362 glacial ice sightings fulfilled this condition, with their derived drift tracks shown in Fig. 3a.

The applied concentration criterion is grounded in an evaluation of the tracking approach against independent drift observations. First, trajectories of 50 GPS-equipped sea ice buoys deployed in the Arctic Ocean between 2012 and 2016 (Meereisportal.de data repository^61^) were reconstructed using IceTrack and directly compared with their observed GPS positions. The mean great-circle deviation between reconstructed and observed locations amounts to approximately 47 ± 48 km after 100 days and 109 ± 68 km after 300 days (Extended Data Fig. 10a), indicating reliable reproduction of large-scale drift pathways under compact pack-ice conditions. To assess tracking performance under looser ice conditions, iceberg drift was additionally evaluated using a small set of GPS-equipped icebergs that were partially exposed to open-water conditions. The applied dataset comprises 11 icebergs tracked between 2012 and 2025 in the Beaufort Gyre and north of the Canadian Arctic Archipelago (data sources: International Arctic Buoy Programme Ice Island Archive, https://iabp.apl.uw.edu/Ice_Islands_2025.html; Carleton University Ice Island Drift Tracking Database, https://wirl.carleton.ca/research/ice/ice-islands/ibtd/). The comparison between reconstructed and observed trajectories indicates larger deviations (55 ± 70 km after 100 days and 286 ± 123 km after 300 days), particularly during periods when sea ice concentration falls below 80% (Extended Data Fig. 10b). Despite the higher deviations under loose ice conditions, positional deviations remain sufficiently small to distinguish iceberg source regions at basin and sector scale, although attribution to individual marine-terminating glaciers is not supported, particularly for trajectories involving extended drift durations.

To assess whether the increasing number of icebergs observed in Fram Strait could, in part, be explained by an enhanced input of glacial ice from the Canadian Arctic, we applied the same Lagrangian tracking framework. Sea ice crossing a virtual gate at 83° N (25° W–25° E) was traced backwards in time using an identical Lagrangian backtracking configuration to that described in ref. ^45^. The Beaufort fraction was defined as the proportion of tracked trajectories that intersected the Beaufort Gyre region prior to export through Fram Strait (Extended Data Fig. 9). Temporal changes in this fraction were analysed to evaluate whether the contribution of Beaufort-origin ice increased after 2000.

### Glacier changes

In this study, we relate the observed increase in iceberg frequency to changes at the contributing marine-terminating glaciers. These changes can be quantified through several complementary approaches that differ in physical meaning, temporal resolution and data requirements. Although the main outlet glaciers in northeast Greenland are comparatively well observed, many marine-terminating glaciers in the Russian High Arctic lack the consistent, long-term datasets required for detailed dynamic assessments^62^.

#### Frontal ablation

Calving is most directly reflected in changes in frontal ablation. Estimates of frontal ablation rely on high-resolution satellite imagery used to track terminus positions over time, typically through manual or semi-automated delineation. For northeast Greenland, these observations document the extensive retreat and fragmentation of the ZI and NG glaciers since the early 2000s (Extended Data Fig. 4, derived from Landsat 5–8 optical imagery), providing an indication of enhanced calving over the past two decades. By contrast, for many Russian High Arctic glaciers, terminus positions have been mapped only episodically or over relatively short time windows^35,39,63^.

#### Ice discharge

A second approach quantifies ice discharge, defined as the flux of grounded ice across a gate near the grounding line. Discharge isolates the dynamic component of glacier change and, over multi-decadal timescales, typically correlates with changes in calving activity. Although discharge is not identical to frontal ablation, increased discharge generally reflects accelerating flow and dynamic thinning that favour enhanced calving. This method requires ice velocity, ice thickness and bedrock topography and is therefore available only on longer timescales for the major outlet glaciers in northeast Greenland. For ZI and NG, we use the discharge estimates in ref. ^64^, which extend back to 1987 and allow quantification of long-term changes in dynamic mass loss. In Fig. 3c, cumulative discharge estimates relative to the stable period from 1985 to 2000 are shown, providing a robust baseline against which the post-2000 increase can be evaluated.

#### Mass-change estimates

**Extended Data Fig. 7:**
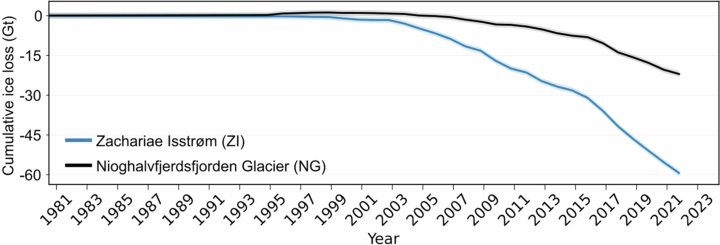
Ice mass loss of NG and ZI over four decades. Time series of ice mass loss of NG and ZI from 1981 to 2022^29,31^.

Where neither frontal-ablation nor discharge records exist, glacier change can be assessed through mass-change estimates derived from remote-sensing products. These measurements reflect the combined effects of surface mass balance and dynamic thinning. For northeast Greenland, we calculate mass changes using NASA Operation IceBridge ATM data and satellite altimetry (ICESat, ICESat-2, CryoSat-2 and ENVISAT), referenced to a 1978 DEM^32,65^. Extended Data Fig. 7 presents the time series of mass loss for the lower sectors of ZI and NG^32^. The two mass-change time series indicate that there was no significant ice loss between 1978 and 2000, an assumption that is also applied when calculating the cumulative discharge shown in Fig. 3c. For SZ and FJL, we rely on published mass-change assessments based on DEM differencing, radar and optical altimetry^36,38,39,62^. Although these studies differ in methodological foundations, resolution and temporal coverage, they consistently report sustained mass loss of marine-terminating glaciers since 2000.

#### Surface velocities

**Extended Data Fig. 8:**
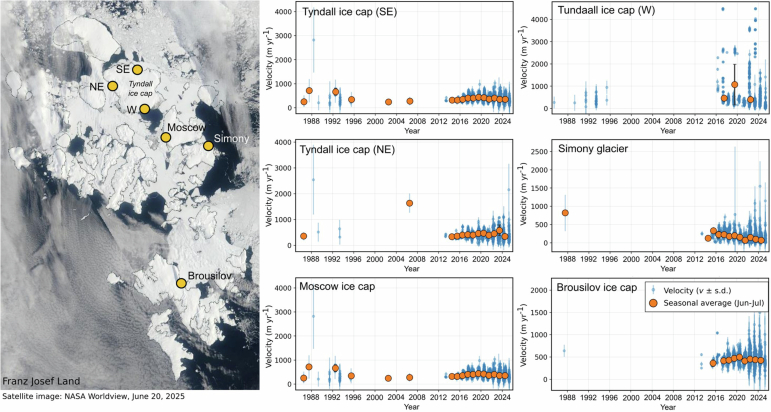
Glacier surface velocity variability on Franz Josef Land. Surface velocity time series for selected marine-terminating glaciers and ice-cap outlets on Franz Josef Land. Individual ITS_LIVE velocity^48^ (*v*) observations derived from Landsat image pairs are shown with associated measurement uncertainties (blue symbols with vertical bars, m yr^−1^ ± s.d.). Seasonal mean velocities (large orange symbols) represent time-weighted averages of observations acquired within ±30 days around 1 July of each year. The satellite image shown on the left was captured by Terra/MODIS on June 20, 2025, and made available through the NASA Worldview application (https://worldview.earthdata.nasa.gov).

**Extended Data Fig. 9:**
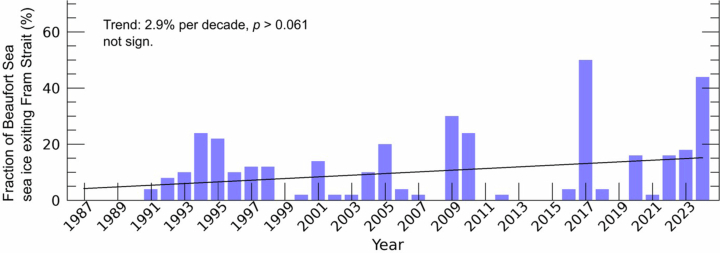
Fraction of Beaufort Sea ice exiting Fram Strait remains stable. Annual fraction (%) of sea ice originating from the Beaufort Gyre and exiting the Arctic Ocean through Fram Strait. Sea ice crossing a virtual gate at 83° N between 25° W and 25° E was backtracked on a monthly basis using IceTrack to determine its region of origin. The methodological framework, including all applied thresholds, follows an identical Lagrangian backtracking configuration to that described in Krumpen et al.^45^. Sea ice was classified as Beaufort-origin if its trajectory crossed 180° longitude south of 85° N and exhibited a minimum drift duration of one year prior to export. No statistically significant increase (black trendline) in the contribution of Beaufort-origin sea ice to Fram Strait export is evident.

**Extended Data Fig. 10:**
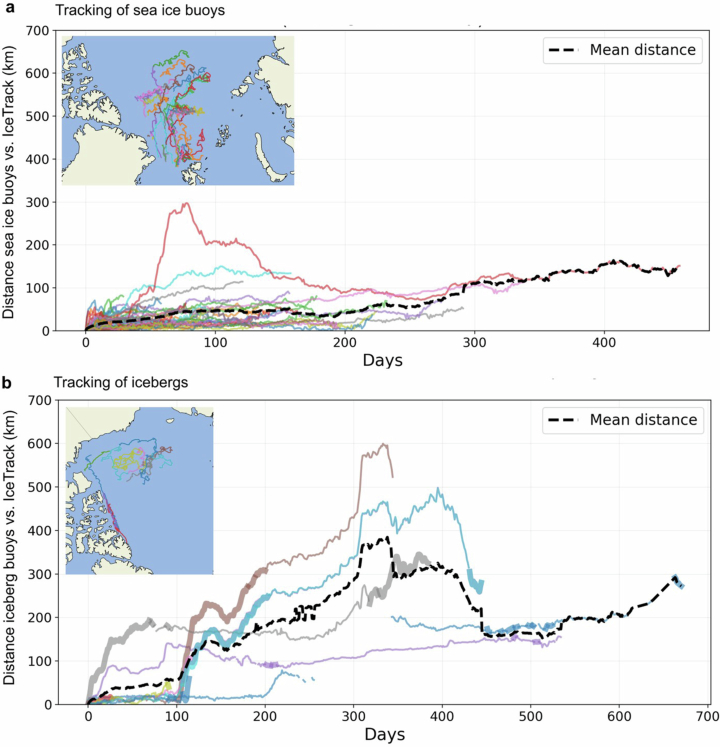
Agreement between observed and reproduced drift trajectories. **a**, Temporal evolution of the distance (km) between the observed drift of sea ice-deployed buoys and the corresponding drift reproduced using the Lagrangian trajectory model IceTrack. The map provides an overview of the drift paths of the buoys used in this analysis. The 50 buoys were deployed between 2012 and 2016 and are available via the Meereisportal.de data repository^61^. **b**, Distance (km) between the observed drift of GPS-equipped icebergs and their reconstructed trajectories obtained with IceTrack. All icebergs considered here were observed within the Beaufort Gyre and north of the Canadian Arctic Archipelago. In total, 11 icebergs equipped with GPS beacons between 2012 and 2025 were analysed. Thick line segments indicate periods during which the sea ice concentration in the vicinity of the icebergs dropped below the threshold of 80% used in this study. Such reduced ice cover is often associated with increased tracking errors (rising distances).

Where direct estimates of frontal ablation or ice discharge are unavailable, glacier dynamics can be assessed using satellite-derived surface velocity time series as indicators of changes in ice flow. We use glacier surface velocities for selected marine-terminating glaciers on FJL and SZ from the NASA MEaSUREs ITS_LIVE project, generated using the auto-RIFT feature-tracking algorithm^48,66^. Each velocity estimate shown in Fig. 4 and Extended Data Fig. 8 represents a temporally averaged surface speed over the image-pair separation interval. Seasonal mean velocities were computed using a moving temporal window centred on 1 July of each year (±30 days). Within each window, velocities were averaged using weights proportional to the image-pair temporal separation to account for unequal temporal coverage.

### FESOM2-iceberg drift model

To quantify potential changes in the large-scale transport of icebergs towards Fram Strait, we conducted a controlled sensitivity experiment using the FESOM2^67^ equipped with an interactive iceberg module^68,69^. The experiment prescribes temporally constant calving fluxes from predefined glacier sectors in NEGIS, FJL, and SZ, allowing us to examine changes in iceberg drift dynamics and associated processes along their trajectories over the simulation period.

FESOM2 is a global, multi-resolution ocean-sea ice model that supports regional mesh refinement in areas of interest. In this study, we use a model configuration with a horizontal resolution of approximately 4.5 km in the Arctic Ocean and Nordic Seas. The model is forced with the JRA-55 atmospheric reanalysis^70^ and has previously been evaluated against observational datasets for Arctic Ocean circulation and sea ice dynamics^71–73^.

The iceberg module represents icebergs as Lagrangian particles with prescribed geometric properties, including height, length and width. These dimensions evolve dynamically during the simulation through thermodynamic processes such as basal and lateral melting, wave-induced erosion and buoyant convection. The momentum balance of model icebergs includes Coriolis and gravitational forces as well as drag forces exerted by the atmosphere, ocean and sea ice. Sea ice drag follows the step-function parameterization described in ref. ^74^. For sea ice concentrations below 15%, sea ice drag is set to zero. When sea ice concentration exceeds 90%, and sea ice strength is greater than 10,000 N m^−3^, icebergs are assumed to be frozen into the pack ice and drift with the surrounding sea ice. A detailed description of the iceberg module is provided in refs. ^68,69^.

In the Southern Ocean, simulated iceberg drift, both in open water and while embedded in pack ice, has been evaluated against observed iceberg trajectories derived from satellite^75,76^ and buoy datasets^77^ in refs. ^68,69^. However, in the Arctic, direct validation is difficult because of the limited availability of observational data. Nevertheless, icebergs that become embedded in dense pack ice are expected to drift largely with the surrounding sea ice cover. Because FESOM2 realistically reproduces observed Arctic sea ice drift and variability^71^, we assume that the drift of icebergs entrained in pack ice is captured realistically by the model.

Whenever the simulated iceberg draft exceeds the local bathymetry, the iceberg is assumed to be grounded. Basal melting continues during grounding and can eventually reduce the draft sufficiently for the iceberg to refloat. Discrete icebergs are generated from an integrated calving flux. The prescribed ice discharge is converted into an equivalent calving area by dividing the flux by the assumed iceberg thickness (250 m; refs. ^78,79^). Individual iceberg sizes are sampled from a −2.2 power-law size distribution following ref. ^80^. Icebergs are assumed to have a quadratic surface and cuboidal geometry. For icebergs smaller than 250 m in horizontal extent, height is set equal to the horizontal dimensions, whereas larger icebergs are assigned a maximum height of 250 m to reduce the risk of instantaneous grounding after release. In an iterative adjustment procedure, iceberg dimensions are adjusted so that the total iceberg volume matches the prescribed integrated discharge.

To isolate transport dynamics from variations in iceberg production, iceberg release was prescribed with temporally constant annual fluxes throughout the simulation period (1970–2023), thereby preventing changes in prescribed calving rates from influencing the simulated iceberg distribution. Icebergs were released continuously throughout the year rather than at a fixed time of release to avoid introducing artificial seasonal biases. Release locations were defined in sufficiently deep offshore waters rather than directly at glacier grounding lines, thereby minimizing artificial grounding effects and reducing the influence of poorly constrained near-coastal processes such as fast-ice storage or mélange-controlled release.

Icebergs were released in three source sectors identified by the backtracking analysis (Fig. 3a): NEG, FJL and SZ. These regions represent the primary sectors from which icebergs may enter Fram Strait. The simulations, therefore, provide a physically consistent large-scale representation of the dominant drift corridors linking these source regions Fram Strait. For the three source regions, the following integrated calving fluxes, averaged over the time period 2000–2010, were prescribed:

- SZ: 3.15 gigatonnes per annum (Gt a^−1^) (ref. ^63^)
- FJL: 8.78 Gt a^−1^(ref. ^63^)
- NEG: 11.15 Gt a^−1^ (ZI^64^); 8.41 Gt a^−1^ (NG^81^); 0.33 Gt a^−1^ (NEG peripheral glaciers^63^).

The first 16 years (1970–1985) of the simulation are considered a spin-up period to reach an equilibrated number of active icebergs, as suggested in ref. ^79^. To separate the effect of changing ocean and sea ice conditions on iceberg drift from the increase in active icebergs during the spin-up phase, the analysis of iceberg dynamics focuses on the period from 1986 to 2023. To quantify spatial patterns of iceberg transport (Fig. 5), we calculate iceberg transit frequency as the relative frequency of iceberg passages through each grid cell over the simulation period.

## Online content

Any methods, additional references, Nature Portfolio reporting summaries, source data, extended data, supplementary information, acknowledgements, peer review information; details of author contributions and competing interests; and statements of data and code availability are available at 10.1038/s41586-026-10630-4.

## Supplementary information

Peer Review File

## Figures and Tables

**Extended Data Table 1:**
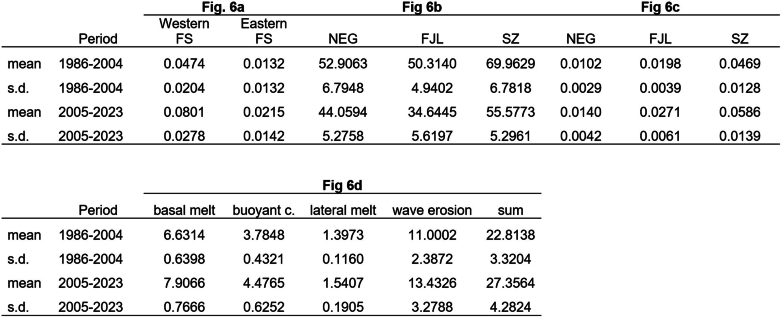
Summary statistics for Fig. 6a–d

Mean and s.d. values for Fig. 6a–d for the two model periods.

## Data Availability

RV *Polarstern* synoptic observations from the past 40 years are available through PANGAEA (10.1594/PANGAEA.987506). All seafloor imagery, including still images and videos collected with towed deep-sea camera systems, is archived in PANGAEA. Dropstone densities, size distributions and associated fauna densities are provided at https://doi.pangaea.de/10.1594/PANGAEA.990270, https://doi.pangaea.de/10.1594/PANGAEA.990271 and https://doi.pangaea.de/10.1594/PANGAEA.990272. Sidescan seafloor data are hosted at https://data.mendeley.com/datasets/2n4rny8xyb/1. Glacier mass-loss and discharge datasets shown in Extended Data Fig. 7 are hosted at Zenodo (https://zenodo.org/records/19007779; ref. ^82^). The data in Fig. 3c are publicly available at 10.22008/promice/data/ice_discharge/d/v02, and FESOM iceberg-drift trajectories and freshwater fluxes are available at Zenodo (https://zenodo.org/records/19067531; ref. ^83^). Data from the NASA ITS_LIVE project can be accessed at the National Snow and Ice Data Center (https://its-live.jpl.nasa.gov/; refs. ^48,66^). Data used for validation of the satellite-based Lagrangian backtracking approach were obtained from the sea ice portal of AWI (https://meereisportal.de), the International Arctic Buoy Programme Ice Island Archive (https://iabp.apl.uw.edu/Ice_Islands_2025.html) and the Carleton University Ice Island Drift Tracking Database (https://wirl.carleton.ca/research/ice/ice-islands/ibtd/). Figures and maps were generated using a combination of Python (v.3.12.9), IDL (v.8.7.3), MATLAB (R2024b and R2025a) and R (v.4.5.1) within RStudio (v.2026.1.0.392). Image processing and figure assembly were carried out using Adobe Photoshop CS6.0, GIMP (v.2.10.34) and Inkscape (v.1.4.2). Geospatial analyses were performed using Python and IDL.

## Code availability

The FESOM2.7 source code used in this study, iceberg input files and mesh information are available at Zenodo (10.5281/zenodo.19661465; ref. ^84^). Documentation of the source code and instructions for running the model are available at https://fesom2.readthedocs.io/en/latest/index.html. Scripts used to produce figures and maps are available at Zenodo (10.5281/zenodo.19664565, ref. ^85^; and 10.5281/zenodo.19667468, ref. ^86^).
