# Supplementary material for: Amplified Arctic iceberg traffic reshapes benthic biodiversity
Source: Nature. 2026 Jun 10;654(8120):963–70. doi: 10.1038/s41586-026-10630-4 (PMC13293891; doi:10.1038/s41586-026-10630-4)
Supplement: Supplementary file 1 — Peer Review File [file 41586_2026_10630_MOESM1_ESM.pdf]

nature portfolio

# Peer Review File

## Amplified Arctic iceberg traffic reshapes benthic biodiversity

Corresponding Author: Dr Thomas Krumpen

Any redactions in this file are there to maintain patient confidentiality, the confidentiality of unpublished data, or to remove third-party material.

**This file contains all reviewer reports in order by version, followed by all author rebuttals in order by version.**

**Attachments originally included by the reviewers as part of their assessment can be found at the end of this file.**

Version 0:

Reviewer comments:

Referee #1

(Remarks to the Author)
Dear Nature Editors and Manuscript Authors,

In this study, Krumpen et al describe an increased accumulation of ice-rafted dropstones in the Fram Strait and link these observations to increased iceberg fluxes from glaciers in the Russian Arctic and North Greenland. Their measurements suggest that these drop stones may reshape benthic habitats in Fram Strait and therefore link observed cryosphere changes with those in the deep ocean. This study is timely and of scientific interest because it details the far-reaching impacts of recent cryospheric changes in our coupled climate system.

In my review for this manuscript, I was asked by the editor to focus on my comments on the glacier discharge and iceberg components of this study. To this end, I had two major comments after reading this manuscript, described here, as well as a number of line and figure suggestions and questions, appended below.

First, the authors use sea ice motion datasets to back-trace observed icebergs to glaciated regions, and conclude that the glaciers in Severnaya Zemlya (SZ) and Franz Josef Land (FJL) are responsible for the majority of icebergs in the Fram Strait region where the drop stones were observed. However, there is no concrete evidence provided in this paper that there has been an increase in calving from these regions, particularly at the timing indicated around the turn of the century. The only information provided is for Greenland glaciers (Figure 3c) but these only make up the majority of the icebergs that drift into the area (line 183). The authors indicate that there is not sufficient data to estimate this effect, but there are hundreds of individual Landsat scenes for this area that do provide a means for estimating changes in ice velocity and retreat. A quick search of the ITS_LIVE dataset (https://its-live.jpl.nasa.gov/ ) shows a collection of these velocity estimates for these regions that have already been processed over the timespan of this study. The velocity estimates are inherently noise, but with large quantities of data, a clear time series like that shown from ZI/79N could be constructed. Providing data from these regions would bolster the argument that the increased iceberg observations after 2000 from the Polarstern do indeed originate in those areas.

Second, the authors use a numerical model run from 1990-2024 with a coupled iceberg drift package to trace iceberg trajectories across the Arctic. However, there is little evidence provided to link the icebergs in this model to the observations in this study, beyond a mean spatial comparison. Some questions that could be quantified from this model that would support the conclusions of this paper are:
- Do the number of icebergs reaching the HAUSGARTEN site in the model increase after 2000?
- Do the relative percentages in iceberg sources (Greenland vs Russian Arctic) in the drop stone area in the model agree with the calculations from sea ice drift?
- What other evidence is there that the model is replicating observed patterns of iceberg drift and decay, particularly in the study region?

Overall, I would recommend this paper for publication in Nature after addressing the major comments above along with the smaller comments below. Please don't hesitate to reach out to me if you have any questions about this review.

Sincerely,

Mike Wood

Line comments:

Line 1: I think the artistic angle of this title leads to misleading interpretations about what the study is about. In particular, the "shadow" of an iceberg doesn't make sense for the study and icebergs themselves are not "rising". Also, I think the word "benthic" or "deep ocean" should be added to qualify "biodiversity"

Line 24: Recommend putting "percent per decade" as d could also mean days.

Line 160 and 161. The first line here describes a trend but then immediately the change is described as a stepwise increase. Looking at the data, it does seem that a stepwise increase occurs, which is not the same thing as a trend. Would recommend quantifying this as a change in the mean before and after a given time (2000?) rather than a trend to better match the data.

Line 177: What is the criteria for trajectory reconstruction? This should be briefly described here.

Line 218: Wood et al 2021

Figure 3: Similar comment to above: given the structure of this data and its description as a step change, I think it would be more appropriate to discuss means before and after 2000. Visually, it doesn't see there is a trend through time, but rather a step change as the authors indicate.

Line 226: I don't think this is true – I think there is data to quantify this change. See major comment above.

Line 264: If the stations are directly in the second pathway from the Russian Arctic, then why even include a discussion of icebergs from ZI and 79N in the main part of this paper?

Referee #2

(Remarks to the Author)
In this study, Krumpen, Meyer-Kaiser and co-authors study how icebergs shed from high Arctic glaciers may transport and deposit ice-rafted debris across the Arctic Basin, and how these processes may have changed over the past decades.

I was impressed with the wealth of innovative ideas and interdisciplinary lines of inquiry the authors present. The manuscript is overall well written and clearly illustrated. However, the individual components strike me as somewhat underdeveloped with each carrying large uncertainties, which taken together makes the conclusions provided rather speculative. As it stands, I don't believe this work is ready for publication.

The most striking shortcoming is the argument (as I understood it) that there has been a shift in drop stone numbers/sizes - however, samples were only collected during two closely-spaced years (2015 and 2017) and changes were apparently not statistically significant (Fig 2b,c), so I wasn't able to follow how this indicated a shift in IRD deposition. The authors themselves state that understanding dropstone changes will require long-term studies (l.122) and I am not sure how the data presented here can support this argument?

Similarly, constraining the iceberg trajectories, either through satellite-derived Lagrangian particle tracking, or through dynamic iceberg modeling is a fascinating topic. But it is really complicated and carries large uncertainties that need to be quantified more carefully before one can put reasonable bounds on the story told here. For example, the fact that after excluding more than 90% of their iceberg sightings for the Lagrangian tracking, the authors still found icebergs originating from unglaciated coastlines made me question the conclusions drawn from this line of inquiry. Similarly, it is unclear how the heat map of iceberg numbers in Fig 4 support the main line of argument presented here. I would think that these iceberg drift investigations should be standalone studies in themselves. Another concern is that icebergs often move relative to the sea ice around it (unless the pack ice is very dense) and there has been some work in this space that I'm not sure was considered (or at least wasn't referenced) - e.g., Lichey and Hellmer (2001). However, it is difficult to assess these impacts since the manuscript doesn't provide sufficient information, and, for example, doesn't provide a discussion how iceberg/sea-ice drag is represented in the FESOM simulations.

Furthermore, there was no discussion of how IRD generally would be distributed in the icebergs and how that impacts the deposition of the IRD - here again is recent work in the literature that would be good considering (e.g., Fendrock et al, 2023, or Meyer et al., 2019). The observations of stones in large numbers on top of the iceberg that was studied in themselves are a fascinating topic that should be explored more carefully (How common is that, how did they get there? What happens when the iceberg capsizes? Etc…) This is particularly striking since a common notion in the iceberg/Heinrich event literature

is that IRD are incorporated into the base of the glacier before icebergs calve, not deposited on their surface.

One more specific comment is that I would argue it's somewhat inconsistent to talk about a step-change in iceberg discharge/presence (around the year 2000), but then present the findings in terms of a linear increase - if it really is a step-change it may make more sense to talk about average numbers before and after the step change.

In summary, my view is that -while intriguing- the arguments laid out in the manuscript do not make a convincing case for the processes and particularly for the changes proposed. More work would need to be done to better constrain the large uncertainties involved.

Lichey, C., & Hellmer, H. H. (2001). Modeling giant-iceberg drift under the influence of sea ice in the Weddell Sea, Antarctica. Journal of Glaciology, 47(158), 452-460.

Fendrock, M., Condron, A., & McGee, D. (2023). Modeling the Production of Heinrich Layers With a Sediment-Enabled Iceberg Model. Paleoceanography and Paleoclimatology, 38(6), e2022PA004583.

Meyer, C. R., Robel, A. A., & Rempel, A. W. (2019). Frozen fringe explains sediment freeze-on during Heinrich events. Earth and Planetary Science Letters, 524, 115725.

Referee #3

(Remarks to the Author)
I have centered my assessment on the ecological aspects, in line with my expertise.

My understanding is that a small-scale image-based analysis of dropstones lying on the seafloor was conducted (at one station, over a single transect of 2.6 km, using a subset of 90 images) and the temporal comparison relied on visual data of stone densities being higher (and stones being more clumped) in 2017 than in 2015 over that same seafloor segment. A few chosen stone-colonizing taxa were also analyzed and their densities shown to follow that of the dropstones.

Overall, I think this is an interesting study that may be worthy of publication; however, the readers deserve more transparency regarding the nature of the evidence and the reasoning. Primarily, it should be made clear what was measured vs what is speculated, i.e. which interpretations are based on actual data (spatial and temporal scales of the physical evidence) vs what was extrapolated to the basin-wide scale in the concurrent analysis of the iceberg frequency/traffic. The fact that more dropstones (colonizing substrates) will reach the seafloor if iceberg traffic increases is intuitive - so the value of this study lies in clarifying the expected delivery patterns and starting to quantify their outcomes.

Importantly, some of the droptsone data processing methodology absolutely needs to be clarified. A clearer acknowledgment of the sources of uncertainty should also be presented with respect to the link established between iceberg frequency and dropstone delivery. Finally, the size distribution of dropstones and how this might play into the colonizing patterns of benthic fauna is currently overlooked (it should ideally be added or else discussed appropriately). I would also argue that the proposed title is a pretty significant leap from the results since "Arctic biodiversity" was not studied here.

I provide some specific comments below (mirrored on the attached PDF file).

L20: I am not sure this adequately describes the results, i.e. higher dropstone density and greater clumping of dropstones detected in one of the two years that were compared at one small specific location of the Fram Strait.

L28: I suggest that "might" reshape would be a more adequate wording.

L65: I think the temporal and spatial framework of the actual study need to be clearly stated here to help readers understand the data-based evidence vs the extrapolations/speculations.

L66: I do not believe the "restructuring" aspect was actually investigated.

L82: How was the glacial origin of the stones confirmed since they were only seen on images and never compared with the debris found in the one iceberg that was examined? Also, what size range of stones was considered (smallest to largest)?

L88: Why and how were images excluded? I could not see any more details in the methods. How data were processed needs to be clarified and justified since much of the whole conclusions (and significance of the study) rely on the comparisons.

L88: Please include the actual density values (and errors) for both years.

L91: Was there any difference in the size distributions of the stones?

L101: Again we need more details and strong justification for this data processing step (outlier corrections).

L102: "two encrusting morphotypes" -- What taxa?

Figure 2 caption L108: Add the size range of the stones? You should explain the elements of the boxplot so the reader can understand the nature of the error bars presented. It would be important to specify the type of fauna that could be counted here, since the study was based on imagery only (there was no collection that would allow a finer analysis or DNA confirmation of identifications). Only one iceberg was sampled? How was the link between the image-based assessments and the nature of the debris found in this iceberg made?

L122: For early colonization patterns, see also Wolvin et al. 2025 in Deep-Sea Res Part II: https://doi.org/10.1016/j.dsr.2025.104454

L125: See the analysis of epibiosis in Wolvin et al. 2025 (PeerJ paper that is already cited).

L244: The increased iceberg frequency seems well supported (though I cannot fully assess this segment) but how this translates into fields of new colonizable debris is a little more obscure (the so called footprint on benthic biodiversity).

L267: But this was not verified with any visual survey. This is deduced by assuming the same patterns would occur globally as the ones seen in the small-scale regional study in 2015 vs 2017?

L287: The nature of this transformation to the deep seafloor is not yet well known and this should be clearly acknowledged.

L647: We would ideally need an analysis of the size of the stones too. This has an impact on the colonizing patterns (and ultimately on the way the stones might impact benthic biodiversity).

L647-648: How was the origin of the stones assessed? Any comparisons with the properties of the stones found in the iceberg (as per above methods)? This comparison has implications for the strength of the link proposed between the iceberg frequency and increased delivery of glacially derived stones to the benthos.

L648: What proportion of the spatial scale under study does this image sub-sampling represent? Because results get extrapolated to basin scale, the reader needs to know the actual scale of the evidence-based study vs the extrapolation (global significance that is brought to light). It is the only way for the reliability of the conclusions to be assessed appropriately.

L650-652: Here we need the details of the limits of the visual detection. What size of taxa could be assessed with certainty?

L655: The main text mentions important steps in the data processing that are not currently justified or described here in the methods. This includes the exclusion of "images with exceptionally high stone densities" (not defined, not justified) and the use of "outlier-corrected dataset" (again not defined and not justified).

Version 1:

Reviewer comments:

Referee #1

(Remarks to the Author)
Dear Manuscript Authors and Nature Editor(s),

This is my second review of Krumpen et al.'s manuscript, "Changing Arctic glaciers amplify iceberg traffic and reshape benthic biodiversity."

In my initial review, I raised two major comments concerning the iceberg calving and drift components of the study. First, I noted that the manuscript did not provide clear evidence that glaciers in the Severnaya Zemlya (SZ) and Franz Josef Land (FJL) regions exhibited increased iceberg flux before versus after the year 2000, despite these areas being identified as the primary source regions for the increased iceberg presence in the eastern dropstone region. Second, I highlighted the lack of comparison between modeled iceberg drift and observational data.

To address the first comment, the authors have made substantial revisions that significantly improve the attribution of increased dropstones to glaciers in the eastern portion of the study area. One of the most compelling additions is Extended Data Figure 8, which shows increased glacier velocities (and thus enhanced iceberg calving) in the SZ region after 2000 relative to earlier periods. This contrasts with minimal changes observed in FJL, and the authors have revised the main text accordingly. These findings are further supported by additional analysis of modeled iceberg drift, demonstrating that the model is able to skillfully reproduce observed iceberg trajectories.

To address the second comment, the authors conducted a sensitivity experiment in which iceberg fluxes are held constant, providing a useful point of comparison. These results are presented in Extended Data Figure 12. This analysis also highlights the role of sea ice and iceberg dynamics in contributing to increased dropstone deposition. Together with the revised glacier flux estimates, this provides a more comprehensive and nuanced assessment of the processes driving iceberg activity in the region, and the manuscript has been updated accordingly.

In addition to these major revisions, the authors have addressed the detailed line comments and made corresponding improvements throughout the manuscript.

After reviewing the revised version, I find that my concerns have been thoroughly addressed. For the components of this study within my area of expertise, I consider this manuscript suitable for publication.

Sincerely,
Mike Wood

Referee #2

(Remarks to the Author)
I'd like to commend the authors for thorough revisions and responses to the reviewer comments. I find the current version much improved. As I said in my original review, the manuscript is full of innovative ideas. My main concerns were that several of the ideas were underdeveloped and that the uncertainties involved were not sufficiently explored. These concerns were mostly allayed for I think two reasons: first, the more detailed explanations and clarifications highlighted that some of my concerns were caused by confusion on my part, and second, the additional results and analysis fill in many of the gaps I saw. However, I still have some general as well as specific comments as detailed below.

General comments:

1) I still struggle to square the conclusion that dropstone density and abundance are statistically significantly higher (l.87/88) with the data as presented in Fig 2b, c. The averages, box, and whiskers, as well as the point clouds in Fig 2b, c seem to be overlapping/not substantively different between 2015 and 2017. Since this is a (the?) key motivating result, I wonder whether the case can be made more convincingly. Maybe one issue is that the metrics chosen to argue for statistical difference in the text (mean plus/minus SE) are different than the box plot metrics in Fig 2? I also struggle with the box plot presentation showing point clouds that have apparently arbitrary horizontal offset. Relatedly, the authors state that outlier images (>5 stones/m^2) were removed (l 781), which seems to clash with the outlier points of ~25 stones/m^2 for 2017 in Fig 2b, and several other values that exceed the 5 stones threshold. Could you present the 2015 and 2017 densities/stone numbers as probability density functions or cumulative distribution functions, maybe on a log-log scale and for example show that the slopes are notably different?

2) The manuscript overall reads somewhat disjointed/scattered. This seems in part a result of the extensive revisions in response to the numerous reviewer comments and in part due to the complex multifaceted scientific approach (with a long methods section and 12 extended data figures). I would urge the authors to careful go over the text and in particular be mindful of what aspects should be in the main text vs methods, and maybe some elements could be streamlined, shortened, or cut.

3) Ice-rafted debris vs dropstones. As far as I could tell, the manuscript does not explicitly distinguish between debris generally vs dropstones, e.g., l 48/49 refers to "ice-rafted dropstones (here meaning debris enclosed in calved icebergs...)". My understanding is that the dropstones which are the focus of this study are of a particular (minimum) size, and distinct from finer debris than can be very abundant. At points (such as l 48/49) the manuscript seems to be using the terms interchangeably. Would it be possible to comment on this minimum size and how it is motivated/justified?

Specific comments:

l 41 A couple more recent citations on increased blooming in response to sea ice changes could be added here, e.g., Ardyna and Arrigo (2020); Castagno et al (2023).

l 49 Maybe just delete "once grounded", since this is a little confusing in this context as icebergs are often referred to as becoming “grounded” but you really mean that the dropstones are "settled on the sea floor".

l 60 "icebergs evade satellite detection" - I would rephrase this to be more specific, since in general there has been a lot of work recently on monitoring icebergs with satellites

l 72 I would maybe delete "primarily concentrated in basal ice layers", since we don't really know whether that's the case?

Fig 2h Would it be insightful to plot the # of stones histogram as pdfs on log-log scale to compare the distributions?

l 138 "sample its entrained lithogenic cargo" This sounds a bit as if you took an ice core and analysed the IRD in that core, whereas, to my understanding, you analysed the dropstones that were found on the iceberg surface. Rephrase to clarify?

l 167 and Fig 3B As with my first-round comment, I don't know how meaningful a linear trend number (and a linear trend line in the figure) is for an evolution that (as the authors argue) exhibits a fairly clear step change around 2000. I would still recommend removing the linear trend line from Fig 3B (and maybe replace with two mean lines for before and after the step change?) The same applies to Ext. Data Fig 3.

Fig 3B I would suggest using different colors for the circles than in Fig 3A - I initially was trying to figure out how the two were related until I realized they weren't.

l 214 maybe delete "... and marks the end of a decades-long quasi stable regime ..." ? I'm not sure there is sufficient evidence for this statement.

Fig 4: What is "iceberg transit frequency" ? Is it different than "iceberg presence"? Maybe define transit frequency?

l 329: I'd suggest adding "[The impact of] accelerating calving ..."

References

- Ardyna, M., & Arrigo, K. R. (2020). Phytoplankton dynamics in a changing Arctic Ocean. Nature Climate Change, 10 (10), 892–903
- Castagno, A. et al (2023). Increased sea ice melt as a driver of enhanced Arctic phytoplankton blooming. Global Change Biology, 29 (17), 5087–5098.

Referee #3

(Remarks to the Author)
Krumpen et al. provided a revised version of their paper retitled “Changing Arctic glaciers amplify iceberg traffic and reshape benthic biodiversity” where they link a localized short-term increase in the accumulation of ice-rafted dropstones in the Fram Strait with increased iceberg traffic coming from glaciers in the Russian Arctic and North Greenland. By assessing faunal colonization of a subset of dropstones, they further suggest that this lithogenic input will reshape benthic habitats in the deep ocean.

I think the authors responded to the concerns of the reviewers in a clear and thorough fashion. The revised version was substantially improved. The analyses were clarified and expanded; some arguments were thereby strengthened while others have now been suitably toned down. I am overall satisfied with the way they addressed the points I had raised (chiefly around benthic ecology), including the need for added details in the methods and results, and the required clarification between evidence-based results and broader inferences. I only have a few minor remarks to make on the revised manuscript:

1. The only sentence I felt might still be a little strong is the one in L327 suggesting the results established a direct connection: “Together, our results establish a direct, climate-driven connection between glacier change at the surface, the passage of icebergs embedded in pack ice, and the resulting increase in available hard-bottom habitats on the deep seafloor.”

2. A question: In the methods L767, the authors state that “OFOS images reliably show seafloor features and fauna >1 cm across”; however, in L754 just above, they indicate “We observed glacial dropstones on the seafloor in OFOS images, ranging 0.2-73 cm across” – so how were stones as small as 0.2 cm reliably measured?

3. For the sake of clarity, I encourage the authors to carefully review the text and make sure citations are placed immediately after the statement they support rather than pooled at the end of a sentence. Some examples:
• L114-116: “Other early colonists of Arctic hard-bottom habitats include hydrozoans 115 and foraminiferans, although these taxa were too small to be detected by the OFOS (Wolvin et al., 116 2025b, Meyer-Kaiser et al, 2019).”
• L122-125: “While empty niche space on newly deposited dropstones is rapidly exploited by early-successional opportunistic species, the assembly of mature communities in polar environments can take decades (Meyer-Kaiser et al., 2025, Dayton et al., 2019; Konar, 2007).”

4. I noted minor style discrepancies in the way figure panels are cited in the captions, e.g. (A), (B)… in Fig 1; bolded A, B,… in Figs 2-3; A), B),… in Fig 4.

Annie Mercier

**Open Access** This Peer Review File is licensed under a Creative Commons Attribution 4.0 International License, which permits use, sharing, adaptation, distribution and reproduction in any medium or format, as long as you give appropriate credit to the original author(s) and the source, provide a link to the Creative Commons license, and indicate if changes were made.
In cases where reviewers are anonymous, credit should be given to 'Anonymous Referee' and the source.
The images or other third party material in this Peer Review File are included in the article's Creative Commons license, unless indicated otherwise in a credit line to the material. If material is not included in the article's Creative Commons license and your intended use is not permitted by statutory regulation or exceeds the permitted use, you will need to obtain permission directly from the copyright holder.
To view a copy of this license, visit https://creativecommons.org/licenses/by/4.0/

Bremen, March 17, 2026

Dear Reviewers,

We thank all three reviewers for their thoughtful and constructive feedback, and appreciate the time and care invested in the reviews.

In the revised manuscript we substantially expanded the methodological description and the discussion of uncertainties. In particular, we (i) expanded the description and validation of the IceTrack back-trajectory approach; (ii) strengthened the assessment of potential Russian Arctic glacier sources by incorporating additional datasets and evaluating alternative source regions; (iii) performed new FESOM simulations to reassess potential changes in iceberg drift patterns; and (iv) moderated the wording of several statements where interpretations previously exceeded the direct evidentiary basis.

We are particularly grateful for the suggestion to further explore the modelling framework. Following this advice, we implemented a revised model configuration in the form of a sensitivity experiment designed to isolate changes in iceberg transport dynamics. The revised simulations indicate that, in addition to intensified calving, the observed increase in iceberg occurrence may also be amplified by more efficient downstream transport associated with a more mobile Arctic sea-ice cover and faster drift speeds. We believe this strengthens the overall interpretation and highlights the link between glacier change, sea-ice dynamics, and deep-sea impacts.

Where revisions involved substantial changes beyond wording adjustments, the modified text is shown in red in the manuscript. Below, reviewer and editor comments are highlighted in blue.

We again thank the reviewers and editors for their constructive comments and hope that the revisions have satisfactorily addressed their concerns.

With best regards,

On behalf of all co-authors

Thomas Krumpen and Kirstin Meyer-Kaiser

## Referee #1 (Remarks to the Author):

*Dear Nature Editors and Manuscript Authors,*

*In this study, Krumpen et al describe an increased accumulation of ice-rafted dropstones in the Fram Strait and link these observations to increased iceberg fluxes from glaciers in the Russian Arctic and North Greenland. Their measurements suggest that these drop stones may reshape benthic habitats in Fram Strait and therefore link observed cryosphere changes with those in the deep ocean. This study is timely and of scientific interest because it details the far-reaching impacts of recent cryospheric changes in our coupled climate system.*

*In my review for this manuscript, I was asked by the editor to focus on my comments on the glacier discharge and iceberg components of this study. To this end, I had two major comments after reading this manuscript, described here, as well as a number of line and figure suggestions and questions, appended below.*

*First, the authors use sea ice motion datasets to back-trace observed icebergs to glaciated regions, and conclude that the glaciers in Severnaya Zemlya (SZ) and Franz Josef Land (FJL) are responsible for the majority of icebergs in the Fram Strait region where the drop stones were observed. However, there is no concrete evidence provided in this paper that there has been an increase in calving from these regions, particularly at the timing indicated around the turn of the century. The only information provided is for Greenland glaciers (Figure 3c) but these only make up the majority of the icebergs that drift into the area (line 183). The authors indicate that there is not sufficient data to estimate this effect, but there are hundreds of individual Landsat scenes for this area that do provide a means for estimating changes in ice velocity and retreat. A quick search of the ITS_LIVE dataset (https://its-live.jpl.nasa.gov/ ) shows a collection of these velocity estimates for these regions that have already been processed over the timespan of this study. The velocity estimates are inherently noise, but with large quantities of data, a clear time series like that shown from ZI/79N could be constructed. Providing data from these regions would bolster the argument that the increased iceberg observations after 2000 from the Polarstern do indeed originate in those areas.*

**Reply:** Thank you for the constructive comment: The linkage between the increased iceberg frequency in the eastern Fram Strait and Russian glaciers is indeed not entirely clear and requires a more in-depth discussion. We address this concern with four additions to the revised manuscript: **(1)** we quantify and report the uncertainty of our sea-ice-based backtracking using independent validation and an explicit uncertainty statement (**new Extended Data Fig. 11**); **(2)** we add and discuss ITS_LIVE velocity for representative outlet glaciers on SZ and FJL (**new Extended Data Fig. 8 and 9**); **(3)** we explicitly evaluate the only other physically plausible non-Greenlandic or Russian Arctic source (the Canadian High Arctic ice shelves/ice islands) and show that its export toward Fram Strait is episodic, rare, and does not exhibit a post-2000 step change (**new Extended Data Fig. 10**); and **(4)** we revise our wording to restrict source attribution to sector scale rather than individual glaciers.

## 1. Uncertainties of backtrajectories

We now provide an explicit assessment of the uncertainty associated with the satellite-based back-trajectory analysis. To evaluate the performance of our tracking approach for sea ice, we first reproduced the trajectories of 50 drifting buoys deployed on the Arctic sea ice and available via the Sea Ice Portal (seaiceportal.de). We focused in particular on buoys deployed within the Transpolar Drift, as this circulation pathway is central to our source-region analysis. For these sea-ice drifters, the mean tracking uncertainty amounts to approximately 47 ± 48 km after 100 days and 109 ± 6.8 km after 300 days, indicating robust performance for reconstructing large-scale drift pathways for icebergs enclosed in pack ice.

Because icebergs - particularly large ones and especially under loose ice conditions - can drift partially independently of the surrounding sea ice, we additionally evaluated the performance of our approach through a reconstruction of iceberg trajectories equipped with GPS beacons. The availability of such iceberg drift datasets in the central Arctic Ocean is limited; nevertheless, we reproduced a small number of published iceberg tracks obtained from https://iabp.apl.uw.edu/Ice_Islands_2025.html and https://wirl.carleton.ca/research/ice/ice-islands/ibtd/. The results show that IceTrack is able to reproduce the general drift behaviour of icebergs, albeit with reduced accuracy compared to standard sea-ice buoys, particularly during periods when icebergs are embedded in loose pack ice (indicated by the thick segments of the trajectories in the new **Extended Data Fig. 9**). For these iceberg tracks, the mean tracking error increases to approximately 55 ± 70 km after 100 days and 286 ± 123 km after 300 days. This validation has been added to the manuscript (see Method Section). It demonstrates why we tracked only a subset of the sightings and explains the choice of the applied threshold values. It also shows that the performance of our tracking approach is sufficient to resolve source regions at basin scale (e.g., distinguishing between Eurasian, Canadian, and Greenlandic sectors), even though it does not allow unique attribution of individual iceberg sightings to specific marine-terminating glaciers.

**Changes made in the manuscript:** We expanded the Methods section (“Backtracking of glacial ice”) to include a quantitative validation of the tracking approach using GPS-equipped sea-ice buoys and icebergs. The limitations of source attribution are now explicitly stated in both the Methods and Results. A new figure (**Extended Data Fig. 11**) presents the validation results.

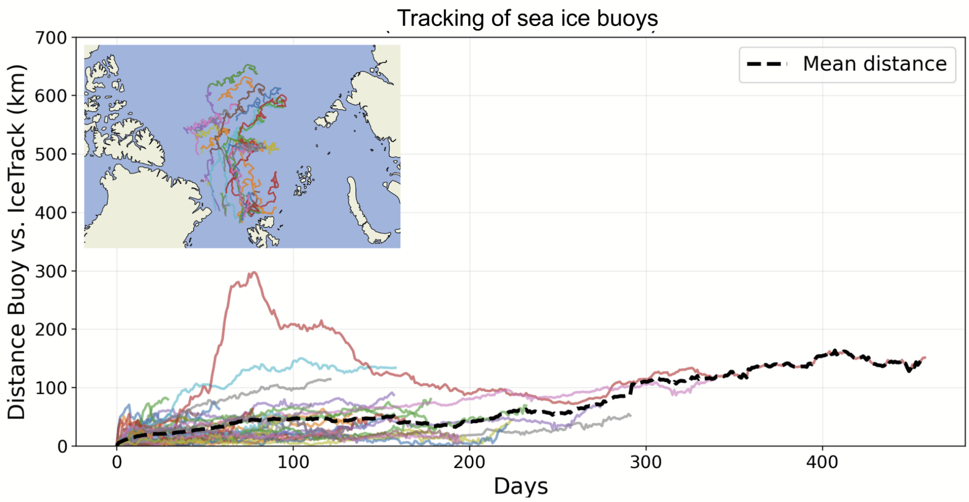


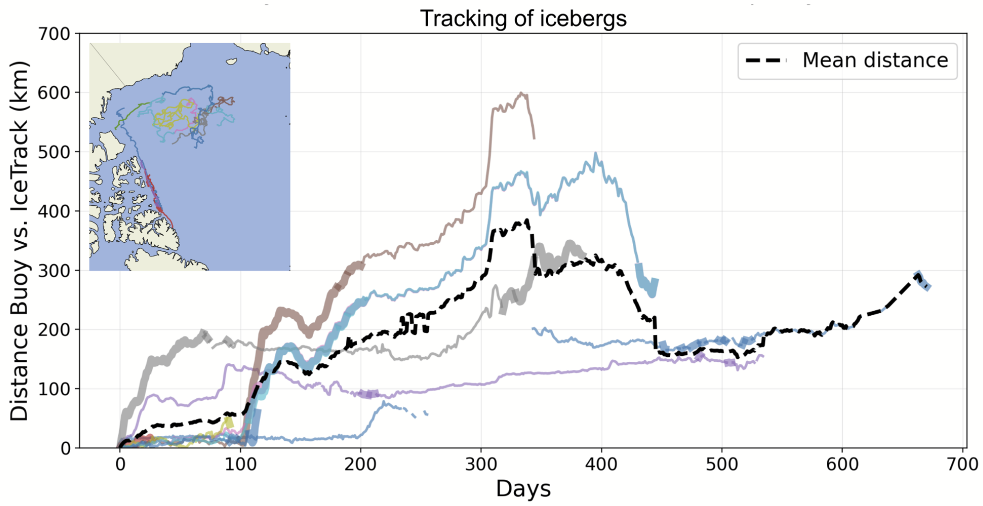


**New Extended Data Fig. 11: Top)** Temporal evolution of the distance (km) between the observed drift of sea-ice-deployed buoys and the corresponding drift reproduced using the Lagrangian trajectory model *IceTrack*. The map provides an overview of the drift paths of the buoys used in this analysis. The 50 buoys were deployed between 2012 and 2016 and are available via the Meereisportal.de data repository. **Bottom)** Distance (km) between the observed drift of GPS-equipped icebergs and their reconstructed trajectories obtained with *IceTrack*. All icebergs considered here were observed within the Beaufort Gyre and north of the Canadian Arctic Archipelago; to our knowledge, a comparable dataset of tracked icebergs within the Transpolar Drift does not exist. In total, 11 icebergs equipped with GPS beacons between 2012 and 2025 were analysed. Thick line segments indicate periods during which the sea-ice concentration in the vicinity of the icebergs dropped below the threshold of 80% used in this study. Such reduced ice cover is often associated with increased tracking errors (rising distances).

## 2. Additional velocity estimate of Eurasian glaciers:

Second, we agree that our initial version did not provide sufficiently direct evidence for a temporal increase in iceberg production from Severnaya Zemlya (SZ) and Franz Josef Land (FJL) around the turn of the century. Following the reviewer's suggestion, we now include satellite-derived ice-flow velocities from the ITS_LIVE dataset for a set of representative outlet glaciers on SZ and FJL (**Extended Data Fig. 8 and 9**). However, as mentioned by the reviewer, the velocity records are noisy and remain fragmented, particularly during the early-2000s transition period, because satellite acquisition and processing efforts at that time were not focused on the Eurasian Arctic. We attempted to account for this limitation by applying appropriate filtering procedures (see the new description in the Methods section).

Accordingly, several outlets on Severnaya Zemlya (Academy of Sciences Ice Cap) exhibit markedly higher flow speeds after 2012 compared to the pre-2000 period. The velocity estimates for glaciers on Franz Josef Land do not show this shift; however, the data coverage there is even more limited. In summary, we interpret the ITS_LIVE results as supportive context indicating enhanced glacier flow on SZ (but not on FJL), although the exact timing of this change cannot be determined due to the gap in the dataset.

**Changes made in the manuscript:** We expanded the Methods ("Surface velocities") to include ITS_LIVE surface velocity time series for representative marine-terminating glaciers on Severnaya Zemlya and Franz Josef Land. Two new figures (**Extended Data Figs. 8 and 9**) present these records. In the revised Results text (line 238-257), we explicitly refer to these figures and state that several outlets on Severnaya Zemlya exhibit higher flow speeds in the post-2012 period compared to the pre-2000 regime, whereas no comparable shift is evident for Franz Josef Land. The velocity records are interpreted as qualitative indicators of glacier-flow changes. Corresponding revisions have also been made to the Abstract to reflect this more cautious interpretation: "In northeast Greenland, the timing of glacier destabilisation and intensified discharge coincides with the rise in iceberg occurrence, whereas sparse satellite coverage in the Russian sector limits precise temporal attribution despite indications of enhanced glacier activity."

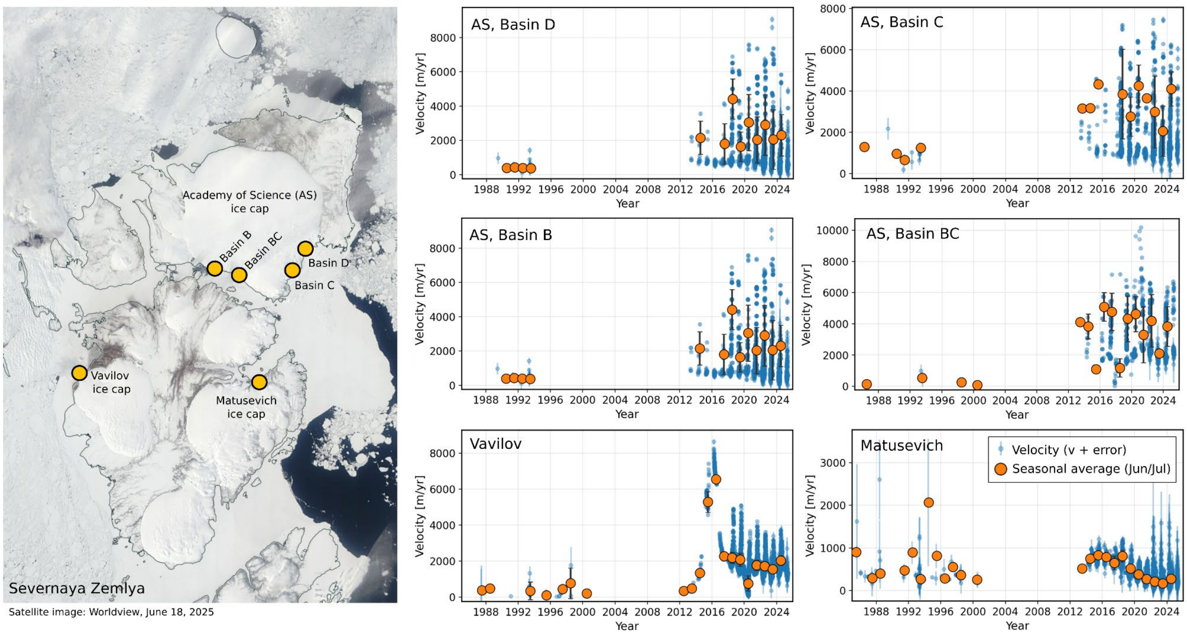


**New Extended Data (Fig. 8): Glacier surface velocity variability on Severnaya Zemlya:** Surface velocity time series for selected marine-terminating glaciers and ice-cap outlets on Severnaya Zemlya. Individual ITS_LIVE velocity observations derived from Landsat image pairs are shown with associated measurement uncertainties (Gardner *et al.*, 2019). Seasonal mean velocities (large symbols) represent time-weighted averages of observations acquired within ±30 days around 1 July of each year.

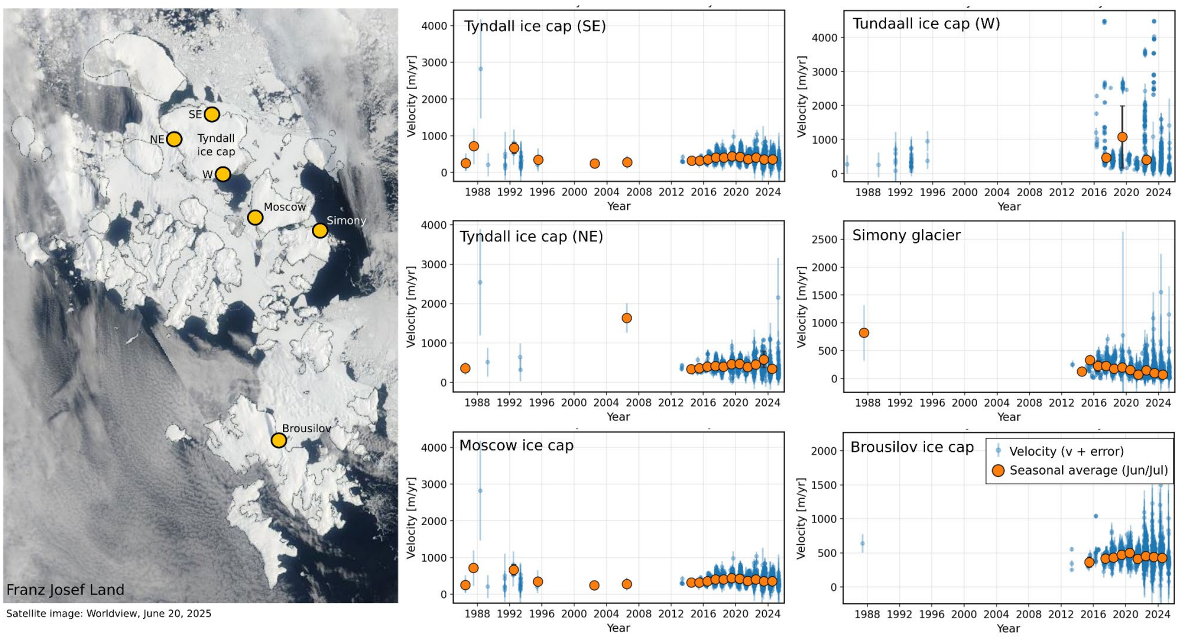


**New Extended Data Fig. 9: Glacier surface velocity variability on Franz Josef Land:** Surface velocity time series for selected marine-terminating glaciers and ice-cap outlets on Franz Josef Land. Individual ITS_LIVE velocity observations derived from Landsat image pairs are shown with associated measurement uncertainties (Gardner *et al.*, 2019). Seasonal mean velocities (large symbols) represent time-weighted averages of observations acquired within ±30 days around 1 July of each year.

## 3. Assessment of alternating sources

Third, we assessed whether an alternative non-Greenlandic or Eurasian source could plausibly explain the stepwise increase observed in the eastern Fram Strait (**Extended Data Fig. 3**). Besides northern Greenland and the Russian High Arctic, the only physically plausible additional Arctic source is the Canadian High Arctic, in particular ice shelves and ice islands originating from Ellesmere Island. Although the Ellesmere ice shelves have experienced substantial disintegration since the early 2000s, drift studies consistently show that ice islands released from these shelves primarily make their way through the Canadian Arctic Archipelago, and only a smaller fraction is transported westward along the Alaskan coast into the Beaufort Gyre. Once incorporated into the Beaufort Gyre, they can circulate there for decades. From there, they may in principle reach Fram Strait via the Transpolar Drift. However, this requires specific and favourable atmospheric forcing and occurs only episodically, as demonstrated by classic cases such as T-3 (van Wychen & Copland, 2017). Other studies likewise indicate that such pathways are rare and that the probability of encountering ice islands far from their source ice shelves is very low (e.g., Fuglem & Jordaan, 2017).

To further exclude the Canadian Arctic Archipelago as a potential contributor to the signal observed in Fram Strait, we explicitly examined the frequency with which Beaufort-Gyre-origin ice reaches Fram Strait. For this purpose, we back-tracked ice exiting Fram Strait at 83° N between 25° W and 25° E on a monthly basis over a period of three decades, using a methodological approach analogous to that described in Krumpen et al. (2019). This analysis clearly (**see new Extended Data Fig. 10**) shows no gradual increase in the occurrence of Beaufort-Gyre-origin ice in Fram Strait. Instead, arrivals are limited to sporadic, episodic events, which were not particularly pronounced during the period between the early 2000s

and 2010. Consequently, an enhanced import of Canadian ice islands into central Arctic Ocean and Fram Strait seems unlikely.

**Changes made in the manuscript:** We added a new subsection in the Methods ("Beaufort-origin sea-ice fraction") describing the Lagrangian analysis of Beaufort-origin sea ice using an identical backtracking configuration to Krumpen et al. (2019). A new figure (**Extended Data Fig. 10**) presents the annual fraction of Beaufort-origin ice exported through Fram Strait. The Results section (line 258-265) has been revised to explicitly evaluate this alternative source hypothesis and to demonstrate that no statistically significant post-2000 increase in Beaufort-origin ice is evident, thereby excluding a Canadian High Arctic source as a plausible driver of the observed stepwise increase in eastern Fram Strait.

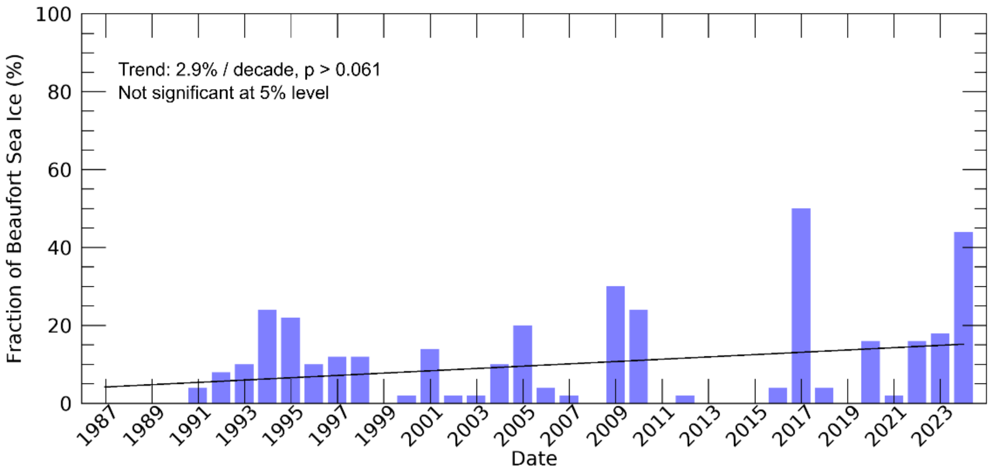


**Extended Data Fig. 10:** Annual fraction (%) of sea ice originating from the Beaufort Gyre and exiting the Arctic Ocean through Fram Strait. Sea ice crossing a virtual gate at 83° N between 25° W and 25° E was backtracked on a monthly basis using IceTrack to determine its region of origin. The methodological framework, including all applied thresholds, follows an identical Lagrangian backtracking configuration to that described in Krumpen et al. (2019). Sea ice was classified as Beaufort-origin if its trajectory crossed 180° longitude south of 85° N and exhibited a minimum drift duration of one year prior to export. No statistically significant increase in the contribution of Beaufort-origin sea ice to Fram Strait export is evident.

**Summary:** We have revised the manuscript's phrasing and logic accordingly. We now avoid implying that the stepwise increase in iceberg frequency in eastern Fram Strait is a direct consequence of intensified calving activity on the Russian side. Instead, we clarify that, based on the available evidence (points 1-3 above), the Eurasian Arctic represents the most plausible additional source region contributing to the eastern Fram Strait signal. However, attribution to specific Russian outlet glaciers remains unresolved and would require targeted reconstructions of frontal retreat and dynamic thinning.

Following the reviewers' helpful comments on the modelling component, we also revisited the model framework to examine whether changes in iceberg transport dynamics could contribute to the signal observed in the eastern Fram Strait. We return to this additional dynamical component in more detail in the responses below when addressing the questions related to the modelling.

***Comment:*** *Second, the authors use a numerical model run from 1990-2024 with a coupled iceberg drift package to trace iceberg trajectories across the Arctic. However, there is little evidence provided to link the icebergs in this model to the observations in this study, beyond a mean spatial comparison. Some questions that could be quantified from this model that would support the conclusions of this paper are:*

*- Do the number of icebergs reaching the HAUSGARTEN site in the model increase after 2000?*

*- Do the relative percentages in iceberg sources (Greenland vs Russian Arctic) in the drop stone area in the model agree with the calculations from sea ice drift?*

**Reply:** Thank you for this helpful suggestion. We agree that the modelling framework offers the opportunity to further explore the observed changes and the processes potentially involved. Following your suggestion, we revised the experimental design of the modelling study, which allowed us to extract additional insights beyond the qualitative comparison presented in the original manuscript. Thanks for pointing this out.

Below we briefly outline the limitations of the model approach, followed by the adjustments made to the experimental design and the additional findings that emerged from the revised analysis.

With respect to the question of how well the model can reproduce absolute changes in iceberg occurrence or their timing, this is inherently difficult. The absolute/relative number of simulated icebergs and their arrival at specific locations depend strongly on how several key processes are parameterized. These include, for example, the prescribed calving fluxes (both magnitude and seasonality), the assumed iceberg size distribution, bathymetry, grounding processes, and the representation of fast-ice formation and breakup. Different reasonable choices for these parameters can lead to substantially different iceberg fluxes. Even with careful parameter selection, simulated iceberg numbers would therefore likely deviate significantly from reality.

We therefore asked ourselves which aspects of the “iceberg system” can be investigated more robustly with the available modelling framework?

The high-resolution, eddy-resolving FESOM configuration realistically represents the large-scale Arctic circulation and sea-ice dynamics; for example, the observed decline in Arctic sea-ice cover after 2006 is well captured by the model (see expanded description of the model in the revised manuscript). In addition, the coupled iceberg module has shown good performance in previous applications in the Antarctic (e.g. Rackow et al., 2017; Ackermann et al., 2024). This provides confidence that the model can realistically represent the dynamical environment controlling iceberg drift (in deeper waters). We therefore focus on the question of whether iceberg drift dynamics may have changed between the two periods. To isolate this dynamical component, we implemented a sensitivity experiment in which calving fluxes (both magnitude and seasonality) are held constant throughout the simulation period (see revised method description of the model). In addition, local shelf processes near the glacier fronts are excluded by releasing the icebergs directly into deeper offshore regions.

We now present these results in a substantially revised final chapter of the manuscript. In this context, **Fig. 4** has been reworked and a **new Extended Data Fig. 12** has been added, which illustrates the processes acting along the iceberg drift pathways. The underlying model analysis is considerably more extensive; however, for the purposes of this study we focus on the key findings most relevant to the interpretation of the observed changes.

**Revised section of the manuscript (line 308 - 368):** While the timing of the iceberg increase in western Fram Strait aligns with changing discharge from the NEGIS outlets, the rise in iceberg occurrence in eastern Fram Strait is not readily attributable to individual glaciers and instead appears to reflect a mixed signal, to which changes in iceberg drift dynamics may also have contributed, albeit with a smaller magnitude.

To examine the dynamic component, we performed a sensitivity experiment with the Finite-VolumE Sea Ice-Ocean Model (FESOM2) coupled to an interactive iceberg module (**see Methods**). Over a 38-year simulation period, icebergs at a constant calving rate were released throughout the year from glaciers in the identified source regions. This configuration is not intended to reproduce the absolute number of icebergs in the Arctic, but rather to isolate the dynamics of drifting icebergs and the processes acting along their trajectories across different phases of the simulation.

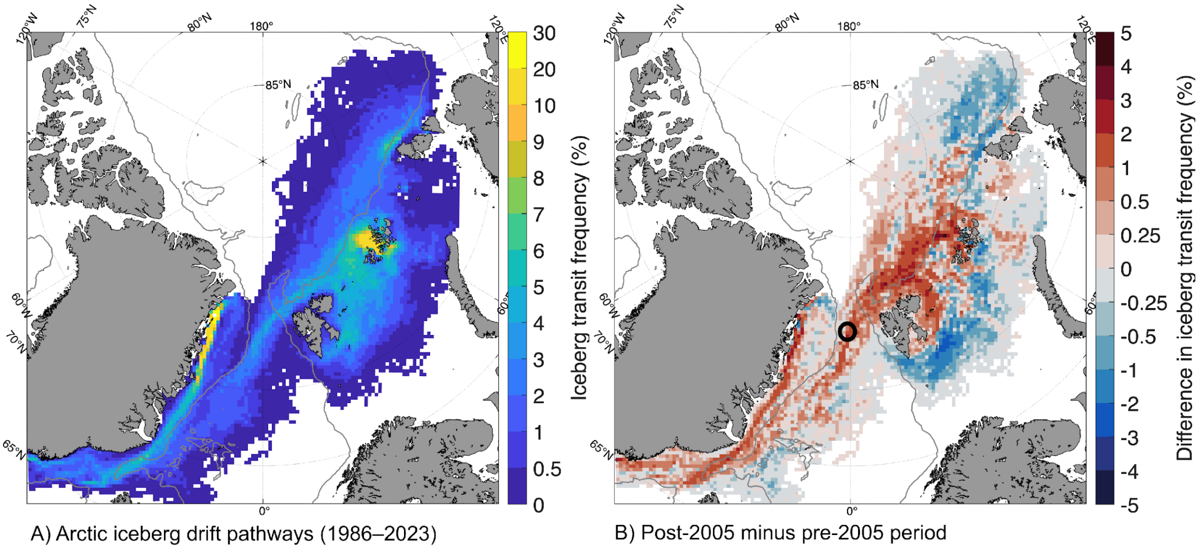


**Fig. 4: Modelled Arctic iceberg drift pathways and their change after 2005.** A) Simulated transit frequency of icebergs released from NG, ZI, SZ and FJL between 1986 and 2023. Two dominant export corridors emerge: a western pathway originating from the NG and ZI glaciers and flowing southward along the East Greenland coast, and an eastern pathway connecting SZ and FJL with central and eastern Fram Strait. B) Difference in transit frequency between the later (post-2005) and earlier (pre-2005) periods. Positive values (red) along both corridors indicate more efficient export toward Fram Strait, whereas negative values (blue) in the Barents and Laptev seas reflect earlier melt under reduced sea ice cover. The black circle shows the position of HAUSGARTEN.

Model results reveal two persistent drift corridors linking the upstream glacier sectors to Fram Strait (**Fig. 4a**). Icebergs released from NG and ZI are transported primarily along the East Greenland margin and enter western Fram Strait, whereas icebergs originating from SZ and FJL are advected across the central Arctic and exit through central and eastern Fram Strait. This spatial structure closely matches the sector-scale source attribution obtained from the satellite-based backtracking analysis and therefore provides independent support for the two dominant export pathways.

Comparing simulated iceberg transit frequencies before and after the stepwise increase around 2005 indicates that both principal pathways experienced a modest but spatially coherent rise in transit frequency in the later period. Although the absolute increase amounts to only a few percentage points, it corresponds to a ~56% increase in the eastern and ~69% in the western Fram Strait relative to baseline frequencies during 1985-2004 (**Extended Data Fig. 12A**). This indicates that the rising Fram Strait iceberg frequencies may arise not only from increased upstream calving but also from more efficient downstream transport. The increase in the number of icebergs crossing western and eastern Fram Strait, together with rising mean drift speeds for icebergs originating from NG, ZI, SZ and FJL is shown in **Extended Data Fig. 12A&C**. The reduced residence time of icebergs reflects the increasing mobility of the surrounding Arctic pack-ice cover, a trend documented in satellite observations (Spreen et al., 2011; Krumpen et al., 2019) and reproduced by the model. Concurrently, the retreating and more dynamic sea ice exposes a larger fraction of icebergs to open-water conditions and winds, reducing the frequency with which they remain embedded in dense pack ice (**Extended Data Fig. 12B**). As a consequence, the simulations show increasing iceberg melt rates, as oceanic warming and wave-induced melt processes become more effective (**Extended Data Fig. 12D**). Together with the pronounced summer sea ice retreat in the Barents and Laptev seas, this leads to a declining iceberg occurrence in these regions (blue shading in **Fig. 4b**).

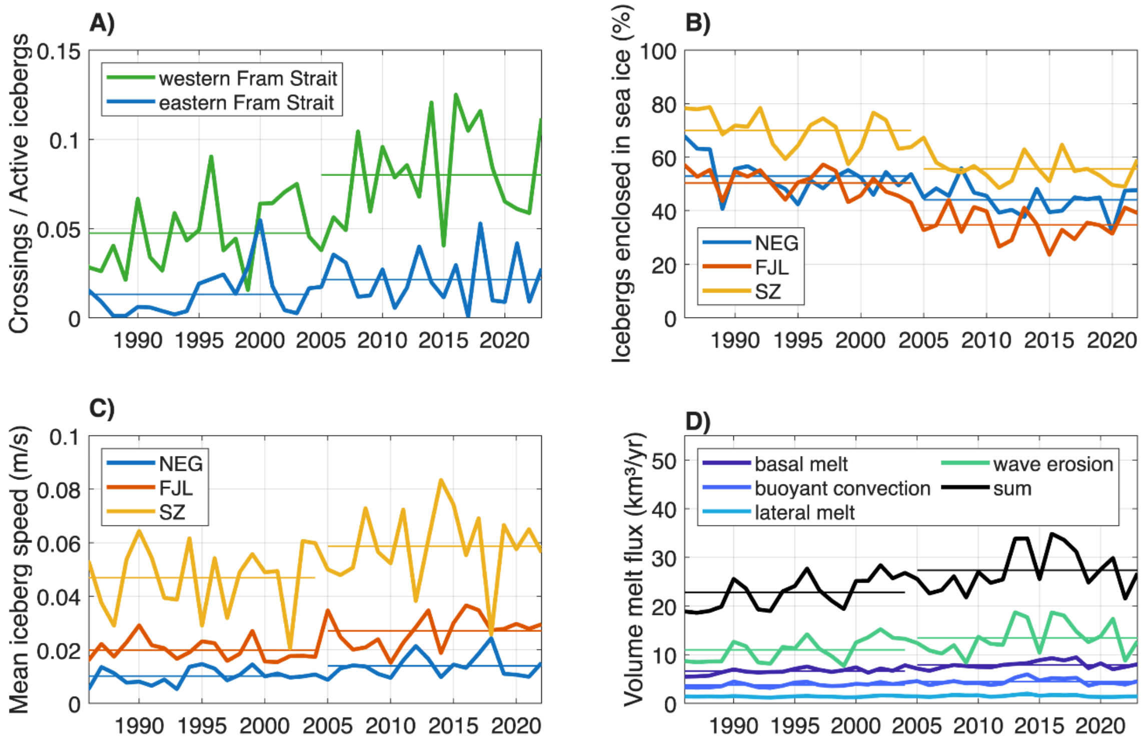


| | | 12A | | 12B | | | 12C | | | 12D | | | | |
|---|---|---|---|---|---|---|---|---|---|---|---|---|---|---|
| | | Western FS | Eastern FS | NEG | FJL | SZ | NEG | FJL | SZ | basal melt | buoyant c. | lateral melt | wave erosion | sum |
| **mean** | **1986-2004** | 0.0474 | 0.0132 | 52.9063 | 50.3140 | 69.9629 | 0.0102 | 0.0198 | 0.0469 | 6.6314 | 3.7848 | 1.3973 | 11.0002 | 22.8138 |
| **sd** | **1986-2004** | 0.0204 | 0.0132 | 6.7948 | 4.9402 | 6.7818 | 0.0029 | 0.0039 | 0.0128 | 0.6398 | 0.4321 | 0.1160 | 2.3872 | 3.3204 |
| **mean** | **2005-2023** | 0.0801 | 0.0215 | 44.0594 | 34.6445 | 55.5773 | 0.0140 | 0.0271 | 0.0586 | 7.9066 | 4.4765 | 1.5407 | 13.4326 | 27.3564 |
| **sd** | **2005-2023** | 0.0278 | 0.0142 | 5.2758 | 5.6197 | 5.2961 | 0.0042 | 0.0061 | 0.0139 | 0.7666 | 0.6252 | 0.1905 | 3.2788 | 4.2824 |

**New Extended Data Fig. 12: Simulated changes in Arctic iceberg transport dynamics.** A) Annual fraction of simulated icebergs crossing virtual gates in western (78°N,25°W-5°W ) and eastern (80°N, 5°W-20°E) Fram Strait (number of crossings relative to the number of active icebergs). B) Mean fraction of simulated icebergs enclosed within dense pack ice during the first year after calving, shown for trajectories originating from NEG, FJL and SZ.

C) Mean iceberg drift speed during the first year after calving for trajectories originating from NEG, FJL and SZ. D) Annual iceberg volume melt flux integrated over all active icebergs in the simulation. Colours show individual melt components (basal melt, buoyant convection, lateral melt and wave erosion), and the black line indicates the total melt flux. The table provides mean and s.d. values for the two periods.

***Comment:*** *What other evidence is there that the model is replicating observed patterns of iceberg drift and decay, particularly in the study region?*

**Reply:** The iceberg model has previously been used and validated for iceberg drift in the Southern Ocean in Rackow et al. (2017) and Ackermann et al. (2024). Modelled iceberg trajectories match satellite and buoy observations reported by Budge and Long (2018) and Stuart and Long (2011). In addition, the revised simulations reproduce the large-scale drift pathways identified in our observational analysis, including the two main transport corridors linking the Greenland and Russian Arctic glacier sectors to Fram Strait.

In the Arctic, observational data on iceberg drift remain sparse. However, icebergs may become locked in thick, concentrated sea ice (Lichey & Hellmer, 2017). When accounting for this lock-in mechanism, as implemented in FESOM, Marson et al. (2024) showed good agreement between modelled iceberg trajectories in Baffin Bay and observations (see revised model description in the Methods section).

***Comment:*** *Overall, I would recommend this paper for publication in Nature after addressing the major comments above along with the smaller comments below. Please don't hesitate to reach out to me if you have any questions about this review.*

**Line comments:**

***Comment:*** *Line 1: I think the artistic angle of this title leads to misleading interpretations about what the study is about. In particular, the "shadow" of an iceberg doesn't make sense for the study and icebergs themselves are not "rising". Also, I think the word "benthic" or "deep ocean" should be added to qualify "biodiversity"*

**Reply:** We agree, may be a bit too artistic. We changed the title to "Changing Arctic glaciers amplify iceberg traffic and reshape benthic biodiversity"

***Comment:*** *Line 24: Recommend putting "percent per decade" as d could also mean days.*

**Reply:** We agree and have replaced the ambiguous "$d^{-1}$" notation. Note that in the revised Abstract and figures, we now report mean annual encounter frequencies before and after the early-2000s breakpoint, thereby explicitly framing the change as a stepwise shift rather than a linear trend. This presentation more accurately reflects the structure of the data and avoids potential misinterpretation

***Comment:*** *Line 160 and 161. The first line here describes a trend but then immediately the change is described as a stepwise increase. Looking at the data, it does seem that a stepwise increase occurs, which is not the same thing as a trend. Would recommend quantifying this as a change in the mean before and after a given time (2000?) rather than a trend to better match the data.*

**Reply:** We agree. We revised the analysis and presentation to treat the change in iceberg encounter rates as an early-2000s regime shift rather than primarily a gradual trend. We now report the mean annual encounter frequency before and after the identified breakpoint, and mention the equivalent linear fit only as a secondary descriptor (with statistical significance and uncertainty). Corresponding text changes were made in the Results and in the figure captions.

***Comment:*** *Line 177: What is the criteria for trajectory reconstruction? This should be briefly described here.*

**Reply:** We have added a brief description of the trajectory-reconstruction criteria directly in the Results at the first mention of the 106/1,362 subset, while keeping the full specification and validation in the Methods. Added: "Trajectories were reconstructed only for sightings embedded in compact pack ice, requiring sea-ice concentration along the back-trajectory to remain ≥80%, with no more than 30 cumulative days below this threshold."

***Comment:*** *Line 218: Wood et al 2021*

**Reply:** Thank you for catching this. We corrected the citation to Wood et al. (2021) and updated the reference list accordingly.

***Comment:*** *Figure 3: Similar comment to above: given the structure of this data and its description as a step change, I think it would be more appropriate to discuss means before and after 2000. Visually, it doesn't see there is a trend through time, but rather a step change as the authors indicate.*

**Reply**: We agree that the early-2000s step change should be made explicit rather than being inferred from a single linear trend. We therefore now highlight pre- and post-2000 mean ± s.d. values in the figure and caption to directly reflect the stepwise nature of the change. The linear fit across the full record is retained for completeness, but it is no longer presented as the primary representation of the signal.

***Comment:*** *Line 226: I don't think this is true - I think there is data to quantify this change. See major comment above.*

**Reply:** The previous wording understated the extent to which glacier-dynamic changes can be evaluated from available satellite observations. In the revised manuscript, we now include ITS_LIVE surface-velocity time series for representative marine-terminating glaciers on Severnaya Zemlya and Franz Josef Land (**Extended Data Figs. 8 and 9**) and explicitly discuss their temporal evolution. These records indicate enhanced flow speeds at several outlets on Severnaya Zemlya, whereas no comparable acceleration signal is evident for Franz Josef Land. We have revised the corresponding text to reflect that dynamic changes can be assessed at the level of individual outlet glaciers, while precise temporal attribution of these changes remains constrained by sparse and irregular satellite coverage in the early-2000s period.

***Comment:*** *Line 264: If the stations are directly in the second pathway from the Russian Arctic, then why even include a discussion of icebergs from ZI and 79N in the main part of this paper?*

**Reply:** Thank you for this comment. Although the HAUSGARTEN stations lie within the eastern drift corridor linking the Russian Arctic to Fram Strait, our observational dataset covers the entire Fram Strait region. The Polarstern observations therefore integrate iceberg occurrences from both sectors. Backtracking indicates that icebergs in western Fram Strait are primarily linked to NE Greenland outlets, while those in eastern Fram Strait are more likely associated with Russian Arctic sources (and potential dynamical changes). We therefore discuss both regions to interpret the overall increase in iceberg occurrence across Fram Strait. The new model results also help to better connect these two regions through the identified drift pathways.

## Referee #2 (Remarks to the Author):

*In this study, Krumpen, Meyer-Kaiser and co-authors study how icebergs shed from high Arctic glaciers may transport and deposit ice-rafted debris across the Arctic Basin, and how these processes may have changed over the past decades.*

*I was impressed with the wealth of innovative ideas and interdisciplinary lines of inquiry the authors present. The manuscript is overall well written and clearly illustrated. However, the individual components strike me as somewhat underdeveloped with each carrying large uncertainties, which taken together makes the conclusions provided rather speculative. As it stands, I don't believe this work is ready for publication.*

**Reply:** We thank the reviewer for this thoughtful assessment of the manuscript and for recognizing the interdisciplinary scope of the study. We agree that several components of the original submission involved uncertainties, and we have therefore revised the manuscript to more clearly document the methods, their limitations, and the level of inference supported by the data.

In particular, we expanded the methodological description and uncertainty assessment of the Lagrangian backtracking approach and now include an explicit validation against independent drift observations (see response to the major comment of Reviewer 1 and new **Extended Data Fig. 11**). In addition, we substantially revised the modelling component. Rather than attempting to reproduce the absolute number of observed icebergs, which depends strongly on uncertain parameters such as calving fluxes and iceberg size distributions, the simulations are now framed as a sensitivity experiment designed to isolate changes in iceberg transport dynamics.

Finally, we moderated the wording throughout the manuscript to more clearly distinguish between observations, interpretations, and hypotheses. Together with the expanded methodological description and the revised model analysis (including the **reworked Fig. 4** and new **Extended Data Fig. 12**), these changes address the reviewer's concerns and strengthen the robustness of the study while preserving its interdisciplinary perspective.

***Comment:*** *The most striking shortcoming is the argument (as I understood it) that there has been a shift in drop stone numbers/sizes - however, samples were only collected during two closely-spaced years (2015 and 2017) and changes were apparently not statistically significant (Fig 2b,c), so I wasn't able to follow how this indicated a shift in IRD deposition. The authors themselves state that understanding dropstone changes will require long-term studies (l.122) and I am not sure how the data presented here can support this argument?*

**Reply:** We thank the reviewer for raising this point. We would first like to clarify that, contrary to the reviewer's interpretation, the differences in dropstone density, dropstone-associated fauna density, and fauna richness are statistically significant (**Fig. 2B-C**). For example, dropstone density increased from 1.65 ± 0.32 stones m$^{-2}$ in 2015 to 2.28 ± 0.31 stones m$^{-2}$ in 2017 ($t_1$ = 6.21, p = 0.01). Importantly, this difference remains significant even after removing outlier images with exceptionally high stone densities, which were excluded to ensure that the results were not driven by a few dense stone clusters. Please see revised method (line 774-783) and manuscript section: "Dropstone density in 2017 (2.28 ± 0.31 stones m$^{-2}$, mean

± SE) was significantly higher than in 2015 (1.65 ± 0.32) ($t_1$ = 6.21, p = 0.01) (**Fig. 2B**), whereas the size distribution of stones differed, and mean stone area decreased from 7.92 ± 1.70 $cm^2$ in 2015 to 4.25 ± 1.96 $cm^2$ in 2017 ($\chi^2$ test, p = 0.003). The distribution of dropstones on the seafloor did not deviate from randomness in 2015 ($\chi^2$ test, p = 0.99), but in 2017 stones were significantly clumped (Morisita index = 1.13, $\chi^2$ test, p < 0.001). To avoid bias from a small number of localized stone clusters, images with exceptionally high stone densities were excluded from the analysis. A similar increase in small dropstones (< 6.4 $cm^2$) was reported in the eastern Fram Strait between 2011 and 2015 (Meyer et al., 2016), suggesting that the observed pattern may extend beyond the single station surveyed here."

At the same time, we agree that the spatial and temporal scope of the observations should be clearly communicated. The seafloor imagery is based on two repeated transects conducted in 2015 and 2017 and therefore documents a short-term, local change in dropstone density only, which we now state explicitly in the manuscript. For example, the Abstract now reads: "Seafloor observations in Fram Strait reveal a short-term localized increase in the density and patchiness of dropstones delivered by debris-laden icebergs." Additional clarifications have been added in the Results section to distinguish between observations and broader interpretations.

The reviewer also refers to our earlier statement regarding long-term studies of dropstone succession. In the revised manuscript we have rephrased this section to avoid confusion between two different processes. Our observations document a short-term change in dropstone deposition, whereas the ecological succession of communities developing on newly deposited stones occurs over much longer timescales (years to decades). The text has therefore been revised to clearly separate these two aspects (line 119-127): "The availability of hard-bottom dropstone habitats is one of the primary drivers of community composition in the deep Arctic Ocean and its influence extends far beyond the stones themselves (MacDonald et al., 2010). Hard-bottom taxa such as sponges, anemones, and soft corals in turn serve as basibionts for epibiotic shrimps, crinoids, and amphipods (Meyer et al., 2016, Wolvin et al., 2025a). Dropstones interrupt laminar flow across the sediment, and the turbulent eddies that result provide heterogeneous habitats that increase meiofaunal diversity (Hasemann et al., 2013). However, while empty niche space on newly deposited dropstones is rapidly exploited by early-successional opportunistic species, the assembly of mature communities in polar environments can take decades (Meyer-Kaiser et al., 2025, Dayton et al., 2019; Konar, 2007)."

***Comment:*** *Similarly, constraining the iceberg trajectories, either through satellite-derived Lagrangian particle tracking, or through dynamic iceberg modeling is a fascinating topic. But it is really complicated and carries large uncertainties that need to be quantified more carefully before one can put reasonable bounds on the story told here. For example, the fact that after excluding more than 90% of their iceberg sightings for the Lagrangian tracking, the authors still found icebergs originating from unglaciated coastlines made me question the conclusions drawn from this line of inquiry.*

**Reply:** We thank the reviewer for raising this important point. We agree that reconstructing iceberg trajectories using either Lagrangian backtracking or numerical modelling is challenging and involves considerable uncertainties. In the revised manuscript we therefore expanded the description of the backtracking approach and its limitations and clarified several aspects of the method.

As explained in the response to Reviewer 1, the strict filtering criteria (≥80% sea-ice concentration along the trajectory, allowing only limited short interruptions) were intentionally applied to ensure that reconstructed trajectories represent icebergs embedded in pack ice and therefore primarily advected by sea-ice drift. This conservative filtering inevitably reduces the number of usable trajectories but increases the robustness of the reconstructed pathways. The choice of this threshold and its implications are now illustrated in the revised manuscript (**new Extended Data Fig. 11**, see also response to reviewer 1).

With respect to trajectories reaching apparently unglaciated coastlines, we clarified that such cases primarily reflect the expected uncertainty associated with very long backtracking times and the limited spatial resolution of the sea-ice drift product near the ice margin (see new text evaluating the performance of IceTrack in the Methods section through comparison with drifters, lines 862-880). As discussed in the revised manuscript, these uncertainties can lead to deviations in the identification of specific glacier sources: "Despite the higher deviations under loose ice conditions, positional deviations remain sufficiently small to distinguish iceberg source regions at basin and sector scale, although attribution to individual marine-terminating glaciers is not supported, particularly for trajectories involving extended drift durations."

Modelling: We agree with the reviewer that modelling iceberg trajectories over basin scales is associated with substantial uncertainties. In particular, absolute iceberg numbers and arrival frequencies depend strongly on poorly constrained parameters such as calving fluxes, iceberg size distributions, and the seasonal timing of iceberg release. Rather than attempting to reproduce these quantities directly, which would require observational constraints that are currently unavailable for the Arctic, we revised the modelling component accordingly.

In the revised manuscript, the model experiments are now framed as a sensitivity study designed to isolate changes in iceberg transport dynamics. By prescribing constant calving fluxes and focusing on the dynamical environment governing iceberg drift, the model can be used more robustly to investigate how changes in ocean and sea-ice conditions affect iceberg transport pathways. We thank the reviewer for this helpful comment, as this revised approach allowed us to identify more efficient downstream transport associated with faster drift conditions, providing an additional dynamical explanation for the observed increase in iceberg delivery to Fram Strait.

In particular, the simulations also reproduce the two main drift corridors identified by the Lagrangian backtracking analysis, which provides confidence in both the observational backtracking approach and the modelling framework. The final chapter of the manuscript has therefore been substantially reworked. Figure 4 has been revised and a new **Extended Data Fig. 12** has been added to illustrate the processes acting along the iceberg drift pathways. We hope that the revised model description and analysis more clearly communicate both the dynamical insights gained from the simulations and the remaining uncertainties of the modelling framework.

***Comment:*** *Similarly, it is unclear how the heat map of iceberg numbers in Fig 4 support the main line of argument presented here. I would think that these iceberg drift investigations should be standalone studies in themselves. Another concern is that icebergs often move relative to the sea ice around it (unless the pack ice is very dense) and there has been some*

*work in this space that I'm not sure was considered (or at least wasn't referenced) - e.g., Lichey and Hellmer (2001). However, it is difficult to assess these impacts since the manuscript doesn't provide sufficient information, and, for example, doesn't provide a discussion how iceberg/sea-ice drag is represented in the FESOM simulations.*

*Bigg, G. R., Wadley, M. R., Stevens, D. P., & Johnson, J. A. (1997). Modelling the dynamics and thermodynamics of icebergs. Cold Regions Science and Technology, 26(2), 113-135.*

*Lichey, C., & Hellmer, H. H. (2001). Modeling giant-iceberg drift under the influence of sea ice in the Weddell Sea, Antarctica. Journal of Glaciology, 47(158), 452-460.*

*Rackow, T., Wesche, C., Timmermann, R., Hellmer, H. H., Juricke, S., & Jung, T. (2017). A simulation of small to giant Antarctic iceberg evolution: Differential impact on climatology estimates. Journal of Geophysical Research: Oceans, 122(4), 3170-3190.*

**Reply:** The results of the newly implemented sensitivity study allow the modelling analysis to be more clearly integrated into the main narrative of the manuscript: The new simulations reproduce the two principal drift corridors that are also identified by the Lagrangian back-trajectory analysis, which increases confidence in both approaches. Furthermore, the sensitivity experiment also highlights that the HAUSGARTEN observatory lies directly within the main transport pathway of icebergs drifting from the Russian High Arctic toward the Arctic outflow. In the previous model configuration, these pathways were less clearly delineated. Moreover, comparing the simulated pathways before and after ~2005 indicates an intensification of downstream transport toward Fram Strait. We clarified this role of the modelling framework in the substantially revised final chapter of the manuscript, including a reworked **Fig. 4** and an expanded model description.

With respect to the iceberg drift in the model (see also revised model description)**:** Thank you for raising this point. The momentum balance of the model icebergs includes Coriolis and gravitational forces as well as drag forces from the atmosphere, ocean, and sea ice. The sea ice force is implemented as a step-function, following the approach by Lichey and Hellmer (2001): for sea ice concentration below 15%, sea ice drag on the icebergs is zero; for sea ice concentration above 90% and sea ice strength above 10,000 N m-1, the icebergs are considered frozen in the sea ice and just move with sea ice velocity. The threshold value of 10,000 N m-1 is smaller than what is considered in Lichey and Hellmer (2001) to account for the sea ice strength being generally lower in our model. For sea ice concentration between 15% and 90% and sea ice strength below 10,000 N m-1, sea ice drag $F_i$ is calculated similar to ocean and atmosphere drag forces:

$$F_i = 0.5 \cdot c_i \cdot \rho_i \cdot A_i \cdot |u_i - u| \cdot (u_i - u)$$

with the drag coefficient $c_i = 1$ after Bigg et al. (1997), $\rho_i = 910\ kg\ m3$, the area applied to sea ice drag $A_i = L \cdot h_i$ with the sea ice thickness $h_i$ and the iceberg and sea ice velocities $u$ and $u_i$. We added some details to the model description section of the manuscript. A detailed model description is given in Rackow et al. (2017).

***Comment:*** *Furthermore, there was no discussion of how IRD generally would be distributed in the icebergs and how that impacts the deposition of the IRD - here again is recent work in the literature that would be good considering (e.g., Fendrock et al, 2023, or Meyer et al., 2019). The observations of stones in large numbers on top of the iceberg that was studied in themselves are a fascinating topic that should be explored more carefully (How common is that, how did they get there? What happens when the iceberg capsizes? Etc…) This is particularly striking since a common notion in the iceberg/Heinrich event literature is that IRD are incorporated into the base of the glacier before icebergs calve, not deposited on their surface.*

**Reply:** Thank you for raising this point. We agree that the previous version did not sufficiently discuss (i) how IRD is typically distributed within glaciers and icebergs and (ii) how this internal debris distribution influences seafloor deposition. We have now expanded the manuscript accordingly. In the revised Introduction and figure caption, we explicitly clarify that IRD is not expected to be uniformly distributed within icebergs nor to accumulate generically at the surface. Instead, debris is primarily concentrated in basal ice facies, consistent with subglacial entrainment processes (e.g., frozen fringe formation; Meyer et al., 2019), and overlain by substantially cleaner meteoric ice. We also revise the schematic to emphasize basal concentration rather than homogeneous debris incorporation.

With respect to our observation of numerous stones on the iceberg surface, we now explicitly state that we cannot determine whether the sampled iceberg had overturned prior to our observations (see chapter: "The Iceberg Conveyor: Cargo, Frequency, and Provenance", and **Fig. Caption 1**). The surface stone accumulations may therefore reflect either (i) supraglacial inheritance from the parent glacier (e.g., medial moraine or rockfall debris), or (ii) post-calving reorientation that exposed debris-rich basal ice at the surface. We make clear in the manuscript that these mechanisms are not mutually exclusive and that the observed surface stones do not contradict the prevailing understanding that IRD is primarily incorporated at the glacier bed.

Finally, we now expand the discussion of how internal stratification governs deposition. Because debris-rich basal layers are located at the base of the iceberg, they typically melt out from the bottom upward unless the iceberg overturns. Vertical gradients in sediment concentration therefore translate into spatially structured rain-out patterns along drift trajectories, with the highest deposition rates expected near the grounding zone and lower-concentration dispersed basal ice melting out farther offshore. Recent sediment-enabled iceberg modeling further demonstrates that predicted Heinrich-layer thickness and extent are sensitive to how sediment is partitioned within icebergs (e.g., concentrated basally versus more distributed) as well as to iceberg size and drift dynamics (Fendrock et al., 2023). Observational constraints from Greenland icebergs (Pierce et al., 2026) further document strong heterogeneity in sediment concentrations and meter-scale debris-rich basal layers, supporting this framework.

See revised last chapter: "We expect that the dispersal of lithogenic material transported by icebergs follows the main drift corridors outlined in **Fig. 4**. The HAUSGARTEN station EG-IV lies directly within the eastern pathway, suggesting that Russian glaciers are a plausible source of the observed dropstone clustering on the seafloor. However, a more precise quantification of dropstone deposition remains challenging because our model does not explicitly simulate

lithic cargo and its release. Dedicated cargo-enabled iceberg models can address this gap, yet their predictive skill still depends on poorly constrained properties of iceberg cargo, including the total lithic load, its vertical distribution within the ice, and how these vary across source regions. Observational constraints from Greenland indicate strong heterogeneity in debris-rich iceberg ice (0.1-45% by mass; median 3.48%, Pierce et al., 2026) and suggest that cargo release is often biased toward early melt-out of basal layers close to the calving front rather than hundreds of kilometres downstream. Comparable constraints for Russian High Arctic sources are currently lacking."

***Comment:*** *One more specific comment is that I would argue it's somewhat inconsistent to talk about a step-change in iceberg discharge/presence (around the year 2000), but then present the findings in terms of a linear increase - if it really is a step-change it may make more sense to talk about average numbers before and after the step change.*

**Reply**: Thank you for this helpful point. We agree that it is inconsistent to describe the change as a regime shift while presenting the results primarily as a linear increase (see also the same comment by Reviewer 1 and our response). We have therefore revised the analysis and presentation to frame the signal as an early-2000s breakpoint and to report mean annual encounter frequencies before and after this breakpoint (with associated uncertainty), rather than emphasizing a single linear trend.

***Comment:*** *In summary, my view is that -while intriguing- the arguments laid out in the manuscript do not make a convincing case for the processes and particularly for the changes proposed. More work would need to be done to better constrain the large uncertainties involved.*

*Lichey, C., & Hellmer, H. H. (2001). Modeling giant-iceberg drift under the influence of sea ice in the Weddell Sea, Antarctica. Journal of Glaciology, 47(158), 452-460.*

*Fendrock, M., Condron, A., & McGee, D. (2023). Modeling the Production of Heinrich Layers With a Sediment-Enabled Iceberg Model. Paleoceanography and Paleoclimatology, 38(6), e2022PA004583.*

*Meyer, C. R., Robel, A. A., & Rempel, A. W. (2019). Frozen fringe explains sediment freeze-on during Heinrich events. Earth and Planetary Science Letters, 524, 115725.*

## Referee #3 (Remarks to the Author):

*I have centered my assessment on the ecological aspects, in line with my expertise.*

*My understanding is that a small-scale image-based analysis of dropstones lying on the seafloor was conducted (at one station, over a single transect of 2.6 km, using a subset of 90 images) and the temporal comparison relied on visual data of stone densities being higher (and stones being more clumped) in 2017 than in 2015 over that same seafloor segment. A few chosen stone-colonizing taxa were also analyzed and their densities shown to follow that of the dropstones.*

*Overall, I think this is an interesting study that may be worthy of publication; however, the readers deserve more transparency regarding the nature of the evidence and the reasoning. Primarily, it should be made clear what was measured vs what is speculated, i.e. which interpretations are based on actual data (spatial and temporal scales of the physical evidence) vs what was extrapolated to the basin-wide scale in the concurrent analysis of the iceberg frequency/traffic. The fact that more dropstones (colonizing substrates) will reach the seafloor if iceberg traffic increases is intuitive - so the value of this study lies in clarifying the expected delivery patterns and starting to quantify their outcomes.*

*Importantly, some of the droptsone data processing methodology absolutely needs to be clarified. A clearer acknowledgment of the sources of uncertainty should also be presented with respect to the link established between iceberg frequency and dropstone delivery. Finally, the size distribution of dropstones and how this might play into the colonizing patterns of benthic fauna is currently overlooked (it should ideally be added or else discussed appropriately). I would also argue that the proposed title is a pretty significant leap from the results since "Arctic biodiversity" was not studied here.*

**Reply**: We thank the reviewer for this careful and constructive assessment. We agree that, in a study combining local seafloor observations with basin-scale iceberg observations, it is essential to clearly distinguish between what is directly measured and what is inferred. We have therefore revised the manuscript throughout to make this distinction more explicit.

First, we now state much more clearly that the seafloor observations document a short-term, localized change at a single HAUSGARTEN station, rather than basin-wide evidence of changing dropstone delivery. The revised Abstract now reads: *"*Seafloor observations in Fram Strait reveal a short-term localized increase in the density and patchiness of dropstones delivered by debris-laden icebergs.*"* We also revised the main text to clarify that the broader spatial interpretation is based on the independent iceberg-frequency record and should be understood as a hypothesis about likely delivery patterns rather than a direct observation of basin-scale dropstone deposition.

Second, we agree that the dropstone data processing required clearer description and justification. We therefore expanded the Methods section to explain the image selection and outlier treatment in detail, including why images with exceptionally high stone densities were excluded and how this step avoids bias from a few localized stone clusters. We also now report the actual density values, associated uncertainties, and the statistical tests used to compare years and quantify clustering.

Third, following the reviewer's suggestion, we added an explicit analysis of dropstone size distribution between 2015 and 2017. This shows that stones were not only more abundant in 2017, but also significantly smaller on average. We further added text to discuss the ecological implications of this result. In particular, we now note that previous work in Fram Strait found no systematic differences in community composition among dropstones of different sizes, suggesting that the observed ecological response is more likely related to increased substrate availability than to size changes alone.

Finally, we have moderated the framing of the manuscript more generally, including revising the title to "Changing Arctic glaciers amplify iceberg traffic and reshape benthic biodiversity", in order to better reflect the scope of the evidence presented.

***More detailed comments:***

***Comment:*** *L20: I am not sure this adequately describes the results, i.e. higher dropstone density and greater clumping of dropstones detected in one of the two years that were compared at one small specific location of the Fram Strait.*

**Reply:** We have updated this sentence to acknowledge that our seafloor observations are short-term and localized, but that trends over broader spatial and temporal scales were drawn from iceberg observations. The text beginning at line 20 now reads: "Seafloor observations in Fram Strait reveal a short-term localized increase in the density and patchiness of dropstones delivered by debris-laden icebergs. At the same time, four decades of shipboard records show that iceberg occurrence increased abruptly in the early 2000s, with mean encounter frequencies rising from 9% (±7.7%) prior to 2000 to 36.3% (±26.4%) thereafter."

***Comment:*** *L28: I suggest that "might" reshape would be a more adequate wording.*

**Reply:** We have added "could" to this line and "may" to line 30: "Along the identified pathways, lithogenic input could reshape deep-sea habitats, and the sharp rise in iceberg frequency may increase navigational hazards as maritime traffic expands in the Arctic"

***Comment:*** *L65: I think the temporal and spatial framework of the actual study need to be clearly stated here to help readers understand the data-based evidence vs the extrapolations/speculations.*

**Reply:** We have added the phrases "short-term" and "one HAUSGARTEN station" to this sentence to be clearer about the data-based evidence we actually have: "In this study, we take a tiered approach that (i) documents a short-term increase in dropstone density and the associated impact on benthic communities at one HAUSGARTEN station…"

***Comment:*** *L66: I do not believe the "restructuring" aspect was actually investigated.*

**Reply:** Thank you for pointing this out. "Restructuring" has been replaced with "imprint on" benthic communities in this sentence (line 69).

***Comment:*** *L82: How was the glacial origin of the stones confirmed since they were only seen on images and never compared with the debris found in the one iceberg that was examined? Also, what size range of stones was considered (smallest to largest)?*

**Reply:** We agree that the origin of the stones and the size range considered should be clarified. We have therefore expanded the Materials and Methods section to explain the reasoning underlying our interpretation that the observed stones represent glacial dropstones (lines 754-764).

In brief, the geological and oceanographic setting of the bathyal Fram Strait strongly limits alternative sources: near-bottom current velocities (<10 cm $s^{-1}$) are insufficient to expose buried lithogenic material, and no bedrock sources occur in the vicinity of station EG-IV. In addition, the lithologies observed on the seafloor (primarily shale and quartz) are consistent with the debris observed on the sampled iceberg (**Extended Data Fig. 2**).

We also added the size range of the stones identified in the imagery. The dropstones observed in OFOS images ranged from 0.2 to 73 cm across, and this information is now reported in the Materials and Methods.

***Comment:*** *L88: Why and how were images excluded? I could not see any more details in the methods. How data were processed needs to be clarified and justified since much of the whole conclusions (and significance of the study) rely on the comparisons.*

**Reply:** We agree that the data processing and image selection required clearer explanation. We have therefore expanded the Methods section to describe in detail how images were screened and why certain images were excluded (line 780). In particular, we now explain that dropstones are often deposited in clusters and that small differences in the swing of the OFOS camera between surveys can cause dense clusters to appear in some images but not others. To avoid biasing the comparison between years by a few localized clusters, we excluded images with exceptionally high stone densities (>5 stones $m^{-2}$). In total, two images were removed from each survey year (2015 and 2017). The revised Methods section now explicitly describes this procedure and its justification.

***Comment:*** *L88: Please include the actual density values (and errors) for both years.*

**Reply**: Thank you for pointing this out. Mean and standard error values for dropstone densities in each year have been added.

***Comment:*** *L91: Was there any difference in the size distributions of the stones?*

**Reply:** Yes. Following the reviewer's suggestion, we added an explicit analysis of the dropstone size distribution between the two survey years. The revised manuscript now reports that the size distribution differed significantly between 2015 and 2017, with the mean stone area decreasing from 7.92 ± 1.70 $cm^2$ in 2015 to 4.25 ± 1.96 $cm^2$ in 2017 ($\chi^2$ test, $p = 0.003$) (line 95).

We also added context by citing a previous study from the eastern Fram Strait that documented a similar increase in small dropstones between 2011 and 2015 (Meyer et al., 2016), suggesting that the observed pattern may extend beyond the single station surveyed here.

***Comment:*** *L101: Again we need more details and strong justification for this data processing step (outlier corrections).*

**Reply:** In line 780, we have explained: "Dropstones are typically deposited on the seafloor in clusters, and slight variations in the swing of the OFOS camera could cause such clusters to appear in one year but not another. To avoid biasing the results by a small number of images containing dense stone clusters, we removed outlier images with anomalously high stone densities (>5 stones $m^{-2}$) prior to analysis (n=2 images removed from each year, 2015 and 2017). "

***Comment:*** *L102: "two encrusting morphotypes" -- What taxa?*

**Reply:** The taxonomic identity of these organisms is unclear because of camera resolution. They are most likely either sponges or bryozoans. This has now been specified in a parenthetical in line 117: "In 2015, all dropstone-associated taxa were randomly distributed ($\chi^2$ test, p>0.05), whereas in 2017, two encrusting morphotypes (likely sponges or bryozoans) and a serpulid polychaete species showed significantly clumped distributions mirroring the dropstone pattern ($\chi^2$ test, p<0.05)."

***Comment:*** *Figure 2 caption L108: Add the size range of the stones? You should explain the elements of the boxplot so the reader can understand the nature of the error bars presented. It would be important to specify the type of fauna that could be counted here, since the study was based on imagery only (there was no collection that would allow a finer analysis or DNA confirmation of identifications). Only one iceberg was sampled? How was the link between the image-based assessments and the nature of the debris found in this iceberg made?*

**Reply:** The size range of the stones visible in panel A has been added to the caption. The elements of the boxplot have been explained in the caption for panel B. The caption now reads: "**Fig. 2:** Dropstone impact on habitat heterogeneity and biodiversity on the seafloor: A, Photo of a large dropstone patch at HAUSGARTEN station EG-IV (2500 m depth, recorded in 2021), with stones 1.2 - 325 $cm^2$; B, boxplot of stone densities in the repeated transect in 2015 and 2017, with the central bar representing the median, box edges representing the interquartile range, and whisker showing quartiles ± 1.5x interquartile range; C, boxplot of the density of dropstone fauna in the repeated transect in 2015 and 2017; D-G, common dropstone taxa; D, unidentified sponges; E, *Tentorium semisuberites*; F, *Bathyphellia margaritacea*; G, *Amphianthus* sp; H, size distributions of stones on the iceberg sampled in 2021 and on the seafloor nearby (station EG-IV, 2500 m depth)."

We have added information to the Materials and Methods (see line 768) describing the fauna that could be reliably visualized in OFOS images and comparison to specimens: "OFOS images reliably show seafloor features and fauna >1 cm across (Purser et al., 2019). Fauna visible in our OFOS images included sponges, bryozoans, tunicates, anemones, soft corals, serpulid polychaetes, barnacles, sea stars, and crinoids. Some taxa could be identified based on taxonomic voucher samples collected in previous studies throughout the Fram Strait (e.g., Bergmann et al., 2009). Taxa for which no voucher had been identified were given morphotype descriptors."

Yes, we only had the opportunity to sample one iceberg in 2021. The link was made between lithogenic material on this iceberg and on the seafloor by comparison of their size distributions (**Fig. 2H**) and visual assessment of their mineral types. We have now explained the logic behind this in the Materials and Methods, line 754: "We observed glacial dropstones on the seafloor in OFOS images, ranging 0.2-73 cm across. The vast majority of stones on the deep seafloor north of 45° N are glacial in origin (Kidd and Huggett 1981). Near-bottom currents in the bathyal Fram Strait are <10 cm $s^{-1}$, not fast enough to uncover buried stones or bedrock (Hasemann et al. 2013, Soltwedel et al. 2013). There is one steep reef in the eastern Fram Strait that experiences swifter near-bottom currents (Meyer et al. 2014). This reef has exposed bedrock, and many stones that have broken off from the reef accumulated at its base (Meyer et al. 2014). However, this reef is hundreds of km from station EG-IV, where our imagery was collected. The stones we observed on the seafloor and stones we observed on the visited iceberg were both shale and quartz (**Extended Data Fig. 2**). Based on the geological, bathymetric, and oceanographic context, stones we observed on the seafloor at station EG-IV must be glacial dropstones."

***Comment:*** *L122: For early colonization patterns, see also Wolvin et al. 2025 in Deep-Sea Res Part II: https://doi.org/10.1016/j.dsr.2025.104454*

**Reply:** Thank you, good hint. This paper has now been cited on line 122.

***Comment:*** *L125: See the analysis of epibiosis in Wolvin et al. 2025 (PeerJ paper that is already cited).*

**Reply:** This citation has been added to line 144.

***Comment:*** *L244: The increased iceberg frequency seems well supported (though I cannot fully assess this segment) but how this translates into fields of new colonizable debris is a little more obscure (the so called footprint on benthic biodiversity).*

**Reply:** We agree that the link between increased iceberg frequency and the supply of colonizable debris on the seafloor should be explained more clearly. Icebergs transport lithogenic material derived from glacier termini and release this debris during drift and melt, resulting in the deposition of dropstones along their trajectories. In the revised manuscript, we therefore clarify that the ecological signal observed at the HAUSGARTEN station reflects the local delivery of such iceberg-derived material rather than a direct basin-wide measurement of debris deposition.

To support this interpretation, we expanded the Materials and Methods section (line 750-759) to explain why the stones observed on the seafloor are interpreted as glacial dropstones. This interpretation is based on the geological and oceanographic setting of the bathyal Fram Strait, which limits alternative sources of lithogenic material, as well as on the lithological similarity between stones observed on the seafloor and debris on the sampled iceberg (**Extended Data Fig. 2**).

***Comment:*** *L267: But this was not verified with any visual survey. This is deduced by assuming the same patterns would occur globally as the ones seen in the small-scale regional study in 2015 vs 2017?*

**Reply:** Yes, it is unfortunately not possible to visually assess dropstone densities across larger areas of the Fram Strait. We have therefore clarified in the revised manuscript that the broader interpretation represents an inference based on the local observations combined with the long-term iceberg record. For added context, we also note in line 99 that "a similar increase in small dropstones (< 6.4 $cm^2$) was reported in the eastern Fram Strait between 2011 and 2015 (Meyer et al., 2016), suggesting that the observed pattern may extend beyond the single station surveyed here."

***Comment:*** *L287: The nature of this transformation to the deep seafloor is not yet well known and this should be clearly acknowledged.*

**Reply:** We agree that the wording "transformation" was too strong. We have therefore replaced this term with "an increase in deep-sea hard-bottom habitats" to more accurately describe the observed effect. In addition, the revised wording now more clearly reflects that the ecological implications of increased dropstone delivery to the deep seafloor remain incompletely understood.

***Comment:*** *L647: We would ideally need an analysis of the size of the stones too. This has an impact on the colonizing patterns (and ultimately on the way the stones might impact benthic biodiversity).*

**Reply:** We agree and therefore have added an analysis of the size distribution of stones in 2015 and 2017 to the manuscript. On line 88, we state: "…,whereas the size distribution of stones differed, and mean stone area decreased from 7.92 ± 1.70 $cm^2$ in 2015 to 4.25 ± 1.96 $cm^2$ in 2017 ($\chi^2$ test, $p = 0.003$)." Regarding biodiversity impacts, we have added on line 104: "Although newly deposited stones were predominantly smaller, previous work in the Fram Strait reported no differences in community composition among dropstones of varying sizes (Meyer et al., 2016), suggesting that the observed size differences are unlikely to influence benthic community composition."

***Comment:*** *L647-648: How was the origin of the stones assessed? Any comparisons with the properties of the stones found in the iceberg (as per above methods)? This comparison has implications for the strength of the link proposed between the iceberg frequency and increased delivery of glacially derived stones to the benthos.*

**Reply:** The origin of the stones was assessed by comparing lithogenic material observed on the sampled iceberg with the stones visible on the seafloor. This comparison is based on both their size distributions (**Fig. 2H**) and their lithological characteristics, which were visually identified as predominantly shale and quartz in both cases.

We have now clarified this reasoning in the Materials and Methods (line 754). In addition, the geological and oceanographic setting of the bathyal Fram Strait strongly limits alternative sources of lithogenic material, supporting the interpretation that the observed stones represent glacial dropstones.

***Comment:*** *L648: What proportion of the spatial scale under study does this image sub-sampling represent? Because results get extrapolated to basin scale, the reader needs to know the actual scale of the evidence-based study vs the extrapolation (global significance that is*

*brought to light). It is the only way for the reliability of the conclusions to be assessed appropriately.*

**Reply:** Thank you for pointing out that this information was missing. We have clarified the spatial extent of the image-based analysis in the revised manuscript. The Methods section now states (line 745):

"In 2015 and 2017, the same transect was surveyed, allowing for a comparison between years in an area of seafloor ~5,400 $m^2$." This value specifies the spatial scale of the direct observations on which the dropstone analysis is based. The broader discussion of iceberg traffic and potential basin-scale implications is now clearly presented as contextual interpretation rather than a direct extrapolation of the image-based observations.

***Comment:*** *L650-652: Here we need the details of the limits of the visual detection. What size of taxa could be assessed with certainty?*

**Reply:** We have added this information in line 763: "OFOS images reliably show seafloor features and fauna >1 cm across (Purser et al., 2019)."

***Comment:*** *L655: The main text mentions important steps in the data processing that are not currently justified or described here in the methods. This includes the exclusion of "images with exceptionally high stone densities" (not defined, not justified) and the use of "outlier-corrected dataset" (again not defined and not justified).*

**Reply:** We have explained the logic and methodology for exclusion of outliers beginning at line 775: "Dropstones are deposited on the seafloor in clusters, and slight variations in the swing of the OFOS camera could lead a cluster of stones to be visible in one year but not another. We did not wish to bias our results by allowing differences between years to be driven by just a few images showing dense clusters of stones. To counteract this, we removed outlier images with anomalously high stone densities (>5 stones $m^{-2}$) prior to analysis (n=2 images removed from each year, 2015 and 2017)."

# Reply to Reviewer

## Reviewer 2

**Comment:** I still struggle to square the conclusion that dropstone density and abundance are statistically significantly higher (l.87/88) with the data as presented in Fig 2b, c. The averages, box, and whiskers, as well as the point clouds in Fig 2b, c seem to be overlapping/not substantively different between 2015 and 2017. Since this is a (the?) key motivating result, I wonder whether the case can be made more convincingly. Maybe one issue is that the metrics chosen to argue for statistical difference in the text (mean plus/minus SE) are different than the box plot metrics in Fig 2? I also struggle with the box plot presentation showing point clouds that have apparently arbitrary horizontal offset. Relatedly, the authors state that outlier images (>5 stones/m^2) were removed (l 781), which seems to clash with the outlier points of ~25 stones/m^2 for 2017 in Fig 2b, and several other values that exceed the 5 stones threshold. Could you present the 2015 and 2017 densities/stone numbers as probability density functions or cumulative distribution functions, maybe on a log-log scale and for example show that the slopes are notably different?

**Reply:** We thank the reviewer for this important comment and agree that the statistical result should be clearly reflected in the data visualization. In response, we explored several alternative representations of the data in **Fig. 2b,c**.

The revised figure now includes mean and standard error values overlaid on the box plots, ensuring consistency with the summary statistics reported in the text. Outliers, which were excluded from the statistical analysis following the reviewer's suggestion in the first round, are retained in the figure to provide a complete and transparent representation of the dataset and are now marked with a distinct symbol. We have also clarified in the figure caption that the horizontal spread of points is arbitrary and used solely to improve visibility.

While alternative representations such as probability density functions can emphasize differences in the distributions (see example below), they do not preserve the visibility of individual observations. Because the statistical conclusions are based on these underlying data points, we consider it important to retain a representation that allows direct inspection of the dataset. The revised **Fig. 2b,c** are shown below, together with a probability density function as an alternative representation.

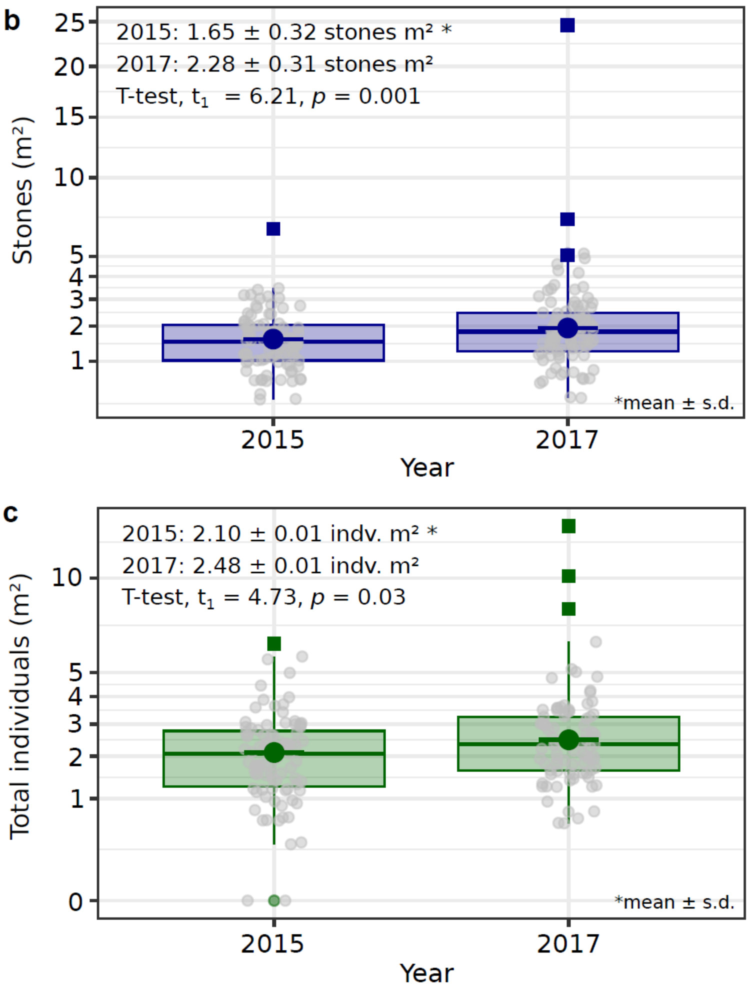


**Fig. 2: Dropstone impact on habitat heterogeneity and biodiversity on the seafloor: […]**; **b**, boxplot of stone densities in the repeated transect in 2015 and 2017, with the central bar representing the median, box edges representing the interquartile range, whiskers showing quartiles ± 1.5x interquartile range, round point representing the mean, error bars representing standard error, gray points representing raw data with arbitrary horizontal spread to aid visualization, and square points representing outliers excluded from statistical analysis; **c**, boxplot of the density of dropstone fauna in the repeated transect in 2015 and 2017; **[…]**.

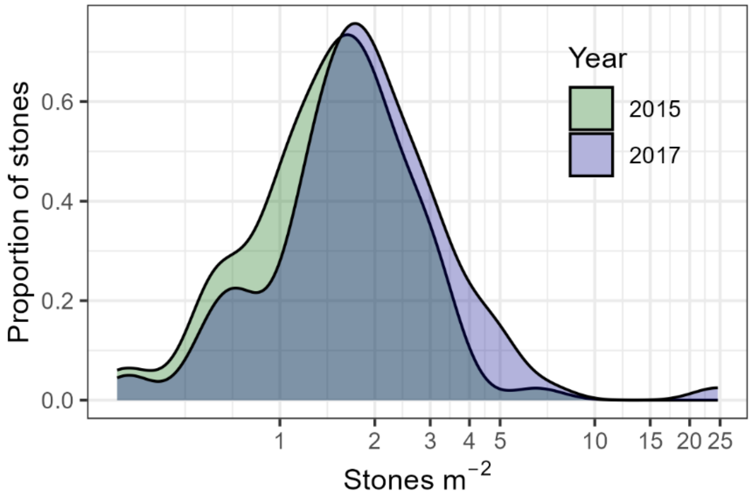


**Figure:** Alternative data representation as a probability density function

**Comment:** The manuscript overall reads somewhat disjointed/scattered. This seems in part a result of the extensive revisions in response to the numerous reviewer comments and in part due to the complex multifaceted scientific approach (with a long methods section and 12 extended data figures). I would urge the authors to careful go over the text and in particular be mindful of what aspects should be in the main text vs methods, and maybe some elements could be streamlined, shortened, or cut.

**Reply:** We appreciate this comment and agree that parts of the manuscript had become somewhat diffuse during revision (e.g. Main and last paragraph). We have therefore carefully streamlined the text, with particular attention to reducing redundancy, and tightening transitions between chapters. Number of ED figures are now limited to 10.

**Comment:** Ice-rafted debris vs dropstones. As far as I could tell, the manuscript does not explicitly distinguish between debris generally vs dropstones, e.g., l 48/49 refers to "ice-rafted dropstones (here meaning debris enclosed in calved icebergs...)". My understanding is that the dropstones which are the focus of this study are of a particular (minimum) size, and distinct from finer debris than can be very abundant. At points (such as l 48/49) the manuscript seems to be using the terms interchangeably. Would it be possible to comment on this minimum size and how it is motivated/justified?

**Reply:** We thank the reviewer for this helpful comment. We have clarified the terminology at the beginning of the manuscript by explicitly distinguishing between ice-rafted debris as the full spectrum of sediment transported by ice (l. 51-53), and its coarse fraction (dropstones), which are the focus of this study. In this context, dropstones refer to the larger clasts that can be reliably identified and quantified in our imagery, distinguishing them from finer debris.

We carefully revised the manuscript to ensure consistent use of these terms, using “dropstones” when referring to the observed and quantified material on the seafloor, and “debris” only in a broader, process-based context.

**Specific comment l 41:** A couple more recent citations on increased blooming in response to sea ice changes could be added here, e.g., Ardyna and Arrigo (2020); Castagno et al (2023).

**Reply:** Thanks for the hint. We replaced Janout et al., 2016 by Ardyna and Arrigo (2020).

**Specific comment l 49:** Maybe just delete "once grounded", since this is a little confusing in this context as icebergs are often referred to as becoming “grounded” but you really mean that the dropstones are "settled on the sea floor".

**Reply:** We agree. “Once grounded” was replaced by “Once deposited”

**Specific comment l 60:** "icebergs evade satellite detection" - I would rephrase this to be more specific, since in general there has been a lot of work recently on monitoring icebergs with satellites

**Reply:** We agree. We changed sentence to "…as smaller icebergs are difficult to detect in satellite observations and leave no long-term record of their abundance" (l 65)

**Specific comment l 72:** I would maybe delete "primarily concentrated in basal ice layers", since we don't really know whether that's the case?

**Reply:** We deleted "primarily concentrated in basal ice layers"

**Specific comment Fig 2h:** Would it be insightful to plot the # of stones histogram as pdfs on log-log scale to compare the distributions?

**Reply:** In response to this comment, we generated probability density functions of stone sizes for both the iceberg and the seafloor (shown below) and explored different axis transformations. However, we found that these representations did not improve the clarity of the comparison. In particular, applying a logarithmic transformation can visually exaggerate differences between the two datasets, whereas statistical testing (chi-square test) indicates no significant difference in their distributions.

We therefore prefer to present the empirical data on untransformed axes.

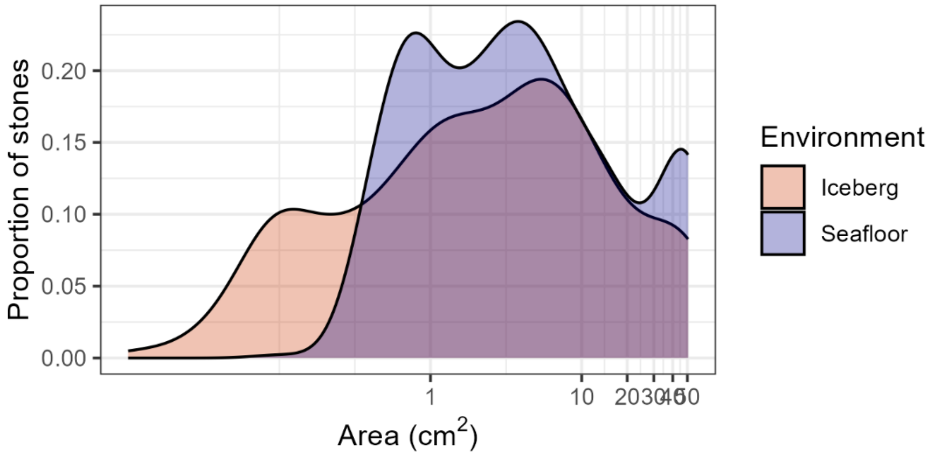


**Figure:** Alternative data representation as a probability density function

**Specific comment l 138:** "sample its entrained lithogenic cargo" This sounds a bit as if you took an ice core and analysed the IRD in that core, whereas, to my understanding, you analysed the dropstones that were found on the iceberg surface. Rephrase to clarify?

**Reply:** Yes, that is somewhat imprecisely phrased. We changed it to "…and collect and analyse stones exposed at its surface".

**Specific comment l 167 and Fig 3B**: As with my first-round comment, I don't know how meaningful a linear trend number (and a linear trend line in the figure) is for an evolution that (as the authors argue) exhibits a fairly clear step change around 2000. I would still recommend removing the linear trend line from Fig 3B (and maybe replace with two mean lines for before and after the step change?) The same applies to Ext. Data Fig 3.

**Reply:** We have removed the linear trend line and now show two lines representing the mean before and after the step change in Fig. 3 and Extended Data Fig. 3.

**Specific comment Fig 3B:** I would suggest using different colors for the circles than in Fig 3A - I initially was trying to figure out how the two were related until I realized they weren't.

**Reply:** We thank the reviewer for pointing this out. We agree that the original color scheme could be misleading. We have therefore revised Fig. 3B to use a distinct color scale, avoiding potential confusion with Fig. 3A.

**Specific comment l 214**: maybe delete "... and marks the end of a decades-long quasi stable regime ..." ? I'm not sure there is sufficient evidence for this statement.

**Reply:** We thank the reviewer for this comment. We agree that the original phrasing was imprecise and could be interpreted as an unsupported conclusion. We have therefore revised the sentence to clarify that this interpretation is based on previous work, and split it into two sentences to improve clarity and attribution (l 91-92)

**Specific comment Fig 4:** What is "iceberg transit frequency" ? Is it different than "iceberg presence"? Maybe define transit frequency?

**Reply:** We agree that the term "iceberg transit frequency" requires clarification. In the revised manuscript, we now define transit frequency as the relative frequency with which simulated icebergs pass through a model grid cell over the course of the simulation. The definition has been added to the caption of Fig. 4 and clarified in the Methods section.

**Specific comment l 329:** I'd suggest adding "[The impact of] accelerating calving ..."

**Reply:** Thank you. "The impact of" was added to the sentence.

## Reviewer 3

**Comment 1:** The only sentence I felt might still be a little strong is the one in L327 suggesting the results established a direct connection: “Together, our results establish a direct, climate-driven connection between glacier change at the surface, the passage of icebergs embedded in pack ice, and the resulting increase in available hard-bottom habitats on the deep seafloor.”

**Reply:** We thank the reviewer for this helpful comment. We agree that the original phrasing may be too strong. We have therefore revised the sentence to more carefully reflect the nature of the evidence and now describe the results as indicating a direct, climate-driven connection rather than establishing it.

**Comment 2:** A question: In the methods L767, the authors state that “OFOS images reliably show seafloor features and fauna >1 cm across”; however, in L754 just above, they indicate “We observed glacial dropstones on the seafloor in OFOS images, ranging 0.2-73 cm across” – so how were stones as small as 0.2 cm reliably measured?

**Reply:** This is a very valid question, and we thank the reviewer for catching the discrepancy. Purser et al. (2019) found that OFOS images reliably detected seafloor organisms and features >1 cm across when images were recorded at 3 m altitude. Our images were recorded at 1.5 m altitude, allowing for visualization of smaller stones. Furthermore, while it is true that the smallest stone dimension was 0.2 cm, this particular stone was longer in its other dimension and anomalously skinny besides. Most of the smallest stones we measured were 0.6 – 0.9 cm across, so it is more accurate to say that the lower detection limit of our survey was 0.6 cm across. We have now updated the figures and added additional explanation to the text, specifying that we could reliably visualize fauna and features to 0.6 cm across (l 644-646).

**Comment 3:** For the sake of clarity, I encourage the authors to carefully review the text and make sure citations are placed immediately after the statement they support rather than pooled at the end of a sentence. Some examples:
• L114-116: “Other early colonists of Arctic hard-bottom habitats include hydrozoans 115 and foraminiferans, although these taxa were too small to be detected by the OFOS (Wolvin et al., 116 2025b, Meyer-Kaiser et al, 2019).”

**Reply:** Both citations have been moved to the middle of the sentence after the word “foraminiferans” for clarity (see line 115).

• L122-125: “While empty niche space on newly deposited dropstones is rapidly exploited by early-successional opportunistic species, the assembly of mature communities in polar environments can take decades (Meyer-Kaiser et al., 2025, Dayton et al., 2019; Konar, 2007).”

**Reply:** We thank the reviewer for this suggestion. In this case, the cited studies support the general statement regarding community assembly times rather than a specific taxon or process within the sentence. We therefore retain the citations at the end of the sentence to reflect their broader relevance.

**Comment 4:** I noted minor style discrepancies in the way figure panels are cited in the captions, e.g. (A), (B)… in Fig 1; bolded A, B,… in Figs 2-3; A), B),… in Fig 4.

**Reply:** We thank the reviewer for pointing this out. This inconsistency was also noted by the editor, and we have revised all figure captions accordingly to ensure a consistent style throughout the manuscript.
